# Oriented Cell Division in the *C*. *elegans* Embryo Is Coordinated by G-Protein Signaling Dependent on the Adhesion GPCR LAT-1

**DOI:** 10.1371/journal.pgen.1005624

**Published:** 2015-10-27

**Authors:** Antje Müller, Jana Winkler, Franziska Fiedler, Tania Sastradihardja, Claudia Binder, Ralf Schnabel, Jana Kungel, Sven Rothemund, Christian Hennig, Torsten Schöneberg, Simone Prömel

**Affiliations:** 1 Institute of Biochemistry, Molecular Biochemistry, Medical Faculty, Leipzig University, Leipzig, Germany; 2 Institute of Genetics, TU Braunschweig, Braunschweig, Germany; 3 Core Unit Peptide Technologies, Medical Faculty, Leipzig University, Leipzig, Germany; University of California San Diego, UNITED STATES

## Abstract

Orientation of spindles and cell division planes during development of many species ensures that correct cell-cell contacts are established, which is vital for proper tissue formation. This is a tightly regulated process involving a complex interplay of various signals. The molecular mechanisms underlying several of these pathways are still incompletely understood. Here, we identify the signaling cascade of the *C*. *elegans* latrophilin homolog LAT-1, an essential player in the coordination of anterior-posterior spindle orientation during the fourth round of embryonic cell division. We show that the receptor mediates a G protein-signaling pathway revealing that G-protein signaling in oriented cell division is not solely GPCR-independent. Genetic analyses showed that through the interaction with a G_s_ protein LAT-1 elevates intracellular cyclic AMP (cAMP) levels in the *C*. *elegans* embryo. Stimulation of this G-protein cascade in *lat-1* null mutant nematodes is sufficient to orient spindles and cell division planes in the embryo in the correct direction. Finally, we demonstrate that LAT-1 is activated by an intramolecular agonist to trigger this cascade. Our data support a model in which a novel, GPCR-dependent G protein-signaling cascade mediated by LAT-1 controls alignment of cell division planes in an anterior-posterior direction via a metabotropic G_s_-protein/adenylyl cyclase pathway by regulating intracellular cAMP levels.

## Author Summary

During embryogenesis an entire organism develops from a single cell. This process is vital for the formation of life, thus cell division occurs with a very distinct orientation and pattern that is tightly controlled by several signaling pathways. The mechanisms underlying these pathways are complex and not yet fully understood. In the roundworm *Caenorhabditis elegans*, a common genetic model, the patterns and orientations in which cells divide in the embryo have been well characterized offering an ideal model to study the molecular mechanisms involved. Here, we show that the signal mediated by the adhesion G protein-coupled receptor LAT-1 is based on cAMP. This second messenger is essential for the orientation of distinct cell division planes in the early embryo. Studies based on a *lat-1* knockout mutant reveal that LAT-1 signaling affects the levels of the second messenger cAMP in the cells via a specific G protein. Thereby the receptor is activated by an intrinsic sequence. This pathway is the first one clearly shown to involve a G protein-coupled receptor-dependent G-protein signal in orientation of embryonic cell division, offering a novel level of regulation of this process among other described pathways.

## Introduction

Spindle and cleavage plane orientation play a central role in many aspects of development as well as homeostasis of organs and organisms. Alignment of spindles during cell division is achieved by interactions of the spindle apparatus with the cell cortex. The machinery linking tubulin to the cortex and supplying force to move the spindle is well characterized [1–3]. However, the molecular mechanisms balancing forces in a specific direction are complex and less well understood. In the early *Caenorhabditis elegans* embryo a large network of only partially characterized signaling pathways including Wnt proteins [4] and PAR proteins [5–7] is engaged in the control of spindle orientations during asymmetric and symmetric cell divisions. These pathways form the basis for the invariant embryonic cell lineage of the nematode with highly reproducible cleavage planes and axes in the early embryo. A sequence of directed cell divisions establishes the particular geometry of early blastomeres, eventually creating an 8-cell stage embryo which contains AB^4^ descendants after the third round of cell cleavage [8]. At this stage, the Wnt/Frizzled (Fz) is involved in spindle rotation of one of the dividing AB descendants to ensure the proper contact of its daughter cells to neighboring blastomeres [9–11]. In the subsequent round of cell division, the Notch pathway induces the left-right asymmetry of the embryo via specific contacts of two of the then eight AB descendants [12,13]. Recently, we have shown that in addition to MOM-2/Wnt and MOM-5/Fz, the latrophilin homolog LAT-1 is a novel receptor required for the coordination of spindles and thus, division plane orientation at the 8-cell stage in the *C*. *elegans* embryo [14]. Some evidence even indicates a function of LAT-1 in parallel to components of the Wnt pathway [14]. In *lat-1(ok1465)* homozygous nematodes the spindle of ABal, one of the AB^4^ cells, is turned almost perpendicular to the anterior-posterior axis in which the cell normally divides. This tilted orientation causes not only one but both daughter cells to touch the neighboring blastomere MS [14] resulting in embryonic lethality as a consequence of incorrect cell-cell contacts. Thus, LAT-1 is involved in orienting cleavage planes in an anterior-posterior direction in descendants of the ABal blastomere. However, it remained elusive how the latrophilin homolog exerts this function.

Latrophilins were initially identified as targets for latrotoxin, a component of the black widow spider´s (*Latrodectus mactans*) toxin [15] and are described as modulators of neurotransmitter release [16,17]. They belong to the class of adhesion G protein-coupled receptors (aGPCRs), a family of seven transmembrane (7TM) receptors with emerging roles in cell and tissue polarity. One of the best understood members of this class, flamingo/CELSR, has been shown to be involved in the planar cell polarity pathway in mice as well as *Drosophila melanogaster* [18,19]. aGPCRs form the second largest class of GPCRs. Despite their essential physiological functions, especially in development and neurobiology [18,20–22], the signaling mechanisms of the majority of these receptors remain poorly understood, and for most family members agonists and downstream effectors are unknown [23,24]. Our previous work showed that LAT-1 mediates two distinct signals, one transduced via the 7TM and the C terminus, while the other requires only the extracellular N terminus containing the GPCR-autoproteolysis inducing (GAIN) domain and its integral motif, the GPCR proteolytic site (GPS) [25], which are characteristic for many aGPCRs [26,27]. During embryonic development, LAT-1 conveys its signal cell autonomously via the 7TM and the C terminus raising essential questions regarding the molecular details and effectors of the signaling cascades triggered by LAT-1.

Here, we have identified the mechanisms of LAT-1 signaling required to mediate the coordination of anterior-posterior tissue polarity in the early *C*. *elegans* embryo. We show that a G_s_ protein-signaling cascade is the key pathway. Following activation by a tethered agonist, LAT-1 elevates levels of the second messenger cyclic AMP (cAMP) via interaction with a G_s_ protein. By increasing intracellular cAMP levels LAT-1 controls spindle orientation in the dividing cell. We discuss how polarity is realized by the respective cascade and the non-polar cAMP signal.

## Results

### LAT-1 signals via the G_s_ protein/adenylyl cyclase/cAMP pathway

**Fig 1:**
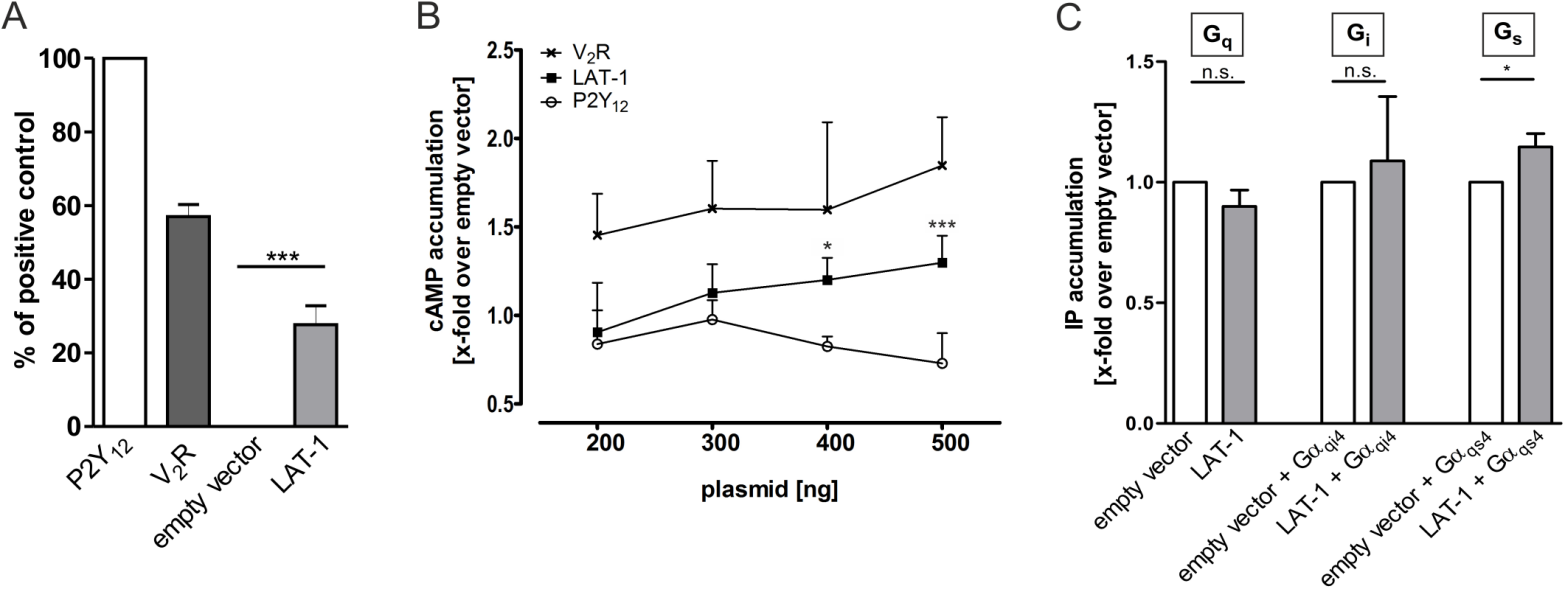
LAT-1 couples to Gα_s_, but not to Gα_i_ or Gα_q_. (A) COS-7 cells were transfected with 500 ng of empty vector (pcDps) or plasmid encoding either human ADP receptor P2Y_12_, the human vasopressin type 2 receptor V_2_R or LAT-1. 48 hours post transfection, surface expression levels were determined with a cell surface ELISA. Expression of the GPCR human vasopressin type 2 receptor V_2_R is shown as a comparison. Data are displayed as percentage of P2Y_12_ (positive control) and given as means ± SD of five independent experiments, each performed in triplicate. The non-specific OD value (empty vector) is 0.02 ± 0.01 (set 0%) and the OD value of P2Y_12_ is 0.95 ± 0.05 (set 100%). *** p < 0.001. (B) To test for functional coupling of LAT-1 to G_s_ proteins, COS-7 cells were transfected with increasing amounts of empty control vector (pcDps) or plasmid encoding LAT-1, human vasopressin type 2 receptor V_2_R, or human P2Y_12_, respectively, and cAMP levels were measured 48 hours later by cAMP accumulation assay. cAMP concentrations are shown as fold change over empty control vector, cAMP levels: 6.2 ± 2.3 nM (200 ng); 6.1 ± 2.5 nM (300 ng); 5.1 ± 2.6 nM (400 ng); 6.1 ± 4.5 nM (500 ng). LAT-1 but not P2Y_12_ causes an increase in cAMP levels. The G_s_-protein coupling V_2_R served as a positive control and the predominantly G_i_-protein coupling P2Y_12_ as negative control. Data are given as means ± SD of five independent experiments, each performed in triplicate. * p < 0.05; *** p < 0.001. (C) For analyses of G_q_-, G_i_- and G_s_-protein coupling, IP accumulation assays were performed detecting Gα_q_-mediated increase in IP levels. To measure functional coupling of LAT-1 to Gα_i_, a chimeric Gα_qi4_ protein was applied to reroute a G_i_-mediated signal to a Gα_q_-mediated pathway. Similarly, to validate G_s_-protein coupling IP accumulation assays were performed using a Gα_qs4_ chimera. For each assay, 1.5 μg of plasmid containing *lat-1* cDNA were transfected (for G_i_- and G_s_-coupling, co-transfection with 100 ng of the respective chimeric protein was applied). No signal was detected for G_q_ or G_i_ protein-signaling pathways, but for G_s_-protein coupling. Basal IP levels are: 220 ± 34 CPM/well (empty vector); 306 ± 20 CPM/well (empty vector + Gα_qi4_); 394 ± 53 CPM/well (empty vector + Gα_qs4_). Data are given as means ± SD of five independent experiments, each performed in triplicate. n.s. not significant; * p < 0.05.

Structure-function analyses have previously shown that the correct alignment of cell division planes of some AB descendants in the early *C*. *elegans* embryo is mediated by the latrophilin homolog LAT-1 via the 7TM-dependent signaling mode of the receptor [14,25]. This could be indicative of a classical GPCR signal via heterotrimeric G proteins. To investigate this hypothesis, we heterologously expressed wild-type *lat-1* in COS-7 cells (Fig 1A and S1 Fig) and tested for functional G-protein coupling of the receptor to the three major G proteins G_s_, G_q_ and G_i_, which are highly conserved between metazoan species [28]. As no interaction partner of LAT-1 has been described which is capable of triggering G protein-mediated signaling of the receptor, its coupling abilities were determined by measuring its basal activity. In the absence of agonists an equilibrium between inactive and active GPCR conformation exists, with only a few receptors residing in the active state [29]. GPCR overexpression increases the amount of receptors in general and thus, in each state. At a certain threshold basal activity of the GPCRs in the active state can be detected and signaling pathways measured, which are normally only activated upon agonist stimulation. The signaling abilities of orphan GPCRs have been frequently identified by taking advantage of this capacity [30,31]. Using this well-established system we first tested the involvement of the G_s_ protein/adenylyl cyclase pathway in LAT-1 signal transduction by measuring the formation of cAMP. Cells overexpressing *lat-1* showed a receptor amount-dependent increase of cAMP levels with a maximum of 1.4-fold (Fig 1B), suggesting that LAT-1 transduces its signal via G_s_ proteins. For the characterization of the G protein-coupling abilities of LAT-1 to G_i_ and G_q_ proteins we performed inositol trisphosphate (IP) accumulation assays. No increase of cellular IP levels was detected (Fig 1C), indicating that LAT-1 does not couple to G_q_ proteins under basal conditions in this assay. It was reported earlier that a chimera, in which the C-terminal 4 amino acids of Gα_i_ (Gα_qi4_) are substituted with the corresponding ones from a Gα_q_ subunit, reroutes the intracellular signals towards a G_q_ pathway modulating IP concentration [32]. Co-expression of *lat-1* with this chimera did not cause an increase of IP concentration, indicating that G_i_-protein coupling appears also improbable (Fig 1C). Applying the same concept by co-expressing a Gα_qs4_ chimera with *lat-1*, which redirects the G_s_ protein signal towards a G_q_-signaling cascade, we detected an increase in basal IP levels when compared to COS-7 cells transfected with empty vector (Fig 1C). By being able to reroute the G_s_ protein signal we verified that the LAT-1-mediated elevation of cAMP levels was due to G_s_-protein coupling and not a result of a secondary effect or Gβγ signaling. Therefore, we conclude that LAT-1 very likely activates the G_s_ protein/adenylyl cyclase signaling pathway.

### Anterior-posterior cell division in the early embryo depends on cAMP-mediated LAT-1 signaling

The question which of the 21 Gα subunits in *C*. *elegans* is activated by LAT-1 is difficult to address as for many of them no effectors or signals are described. One likely candidate is GSA-1, the closest homolog of Gα_s_ in *C*. *elegans* displaying 66% identity to mammalian Gα_s_ proteins [33], which is also expressed in embryos [34]. *gsa-1(pk75)* homozygous animals survive embryogenesis but arrest in larval development [34], which may be explained by a maternal contribution of the embryonic functions. To elucidate a possible interaction of *lat-1* with *gsa-1* in early embryonic development we knocked down maternally and zygotically contributed *gsa-1* activity using RNA interference (RNAi).

**Fig 2:**
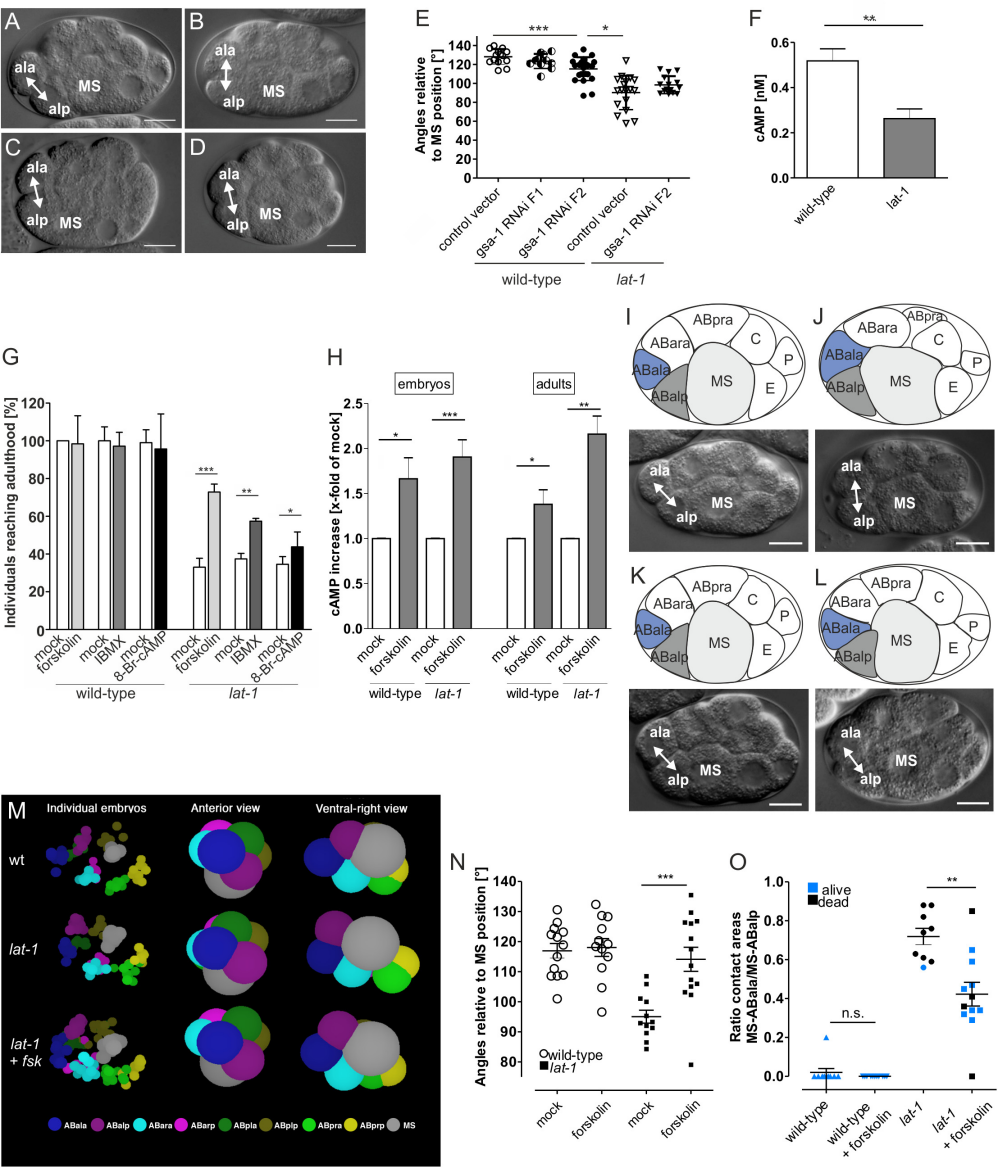
Division plane defects in *lat-1* mutant embryos are rescued by elevation of intracellular cAMP via a G_s_ protein/adenylyl cyclase pathway. (A-D) *gsa-1* RNAi in AB^4^ embryos reveals a similar phenotype to that of *lat-1*, scale bars = 10 μm. In wild-type embryos treated with the RNAi control vector L4440 ABal divides in an anterior-posterior direction (A) whereas in treated *lat-1* mutants the division plane is almost perpendicular to MS (B). *gsa-1* RNAi by feeding over two generations leads to a turning of the F2 ABal division plane in a direction similar to that in *lat-1* mutants (C). F2 *lat-1; gsa-1(RNAi)* embryos show a similar ABal division plane to *lat-1* mutants (D). (E) Cell division plane angles of ABal relative to MS after knockdown of *gsa-1*. Feeding of *gsa-1* RNAi on wild-type nematodes resulted in an ABal division plane angle significantly different to the one of control vector-fed wild-type embryos in the F2 generation. Embryos of the F1 generation did not show a significant different angle. Knockdown of *gsa-1* in *lat-1* mutants in the F2 generation did not change division plane angles. The vector L4440 served as negative control. Data are shown as means ± SD. * p < 0.05; *** p < 0.001; n ≥ 11. (F) cAMP levels are reduced in *lat-1* embryos compared to a control wild-type population. cAMP levels were measured after lysis (15 μg protein) by cAMP accumulation assay. Assays were performed in three independent experiments, data are given as means ± SD. ** p < 0.01. (G) Rescue of developmental lethality in *lat-1* nematodes treated with compounds promoting cAMP accumulation: forskolin (80 μM), IBMX (10 mM) and 8-Br-cAMP (0.25 mM). Mothers and subsequently offspring of wild-type and *lat-1* individuals were incubated and individuals reaching adulthood were scored (n = 410). As controls, nematodes were incubated in solvent without drug (mock). Data are shown as means ± SD. * p < 0.05; ** p < 0.01; *** p < 0.001. (H) Forskolin increases cAMP levels in *C*. *elegans* embryos and adults. *lat-1* mutants and subsequently embryos were incubated with 80 μM forskolin. Embryos displayed an increase in cAMP levels compared to control animals. Similarly, mutant individuals at the L4 stage also showed an elevation in cAMP concentration. cAMP levels were measured after lysis by cAMP accumulation assay. As controls, nematodes were treated with 0.8% DMSO lacking forskolin (mock). Basal cAMP levels of mock control are: 0.70 ± 0.14 nM (wild-type embryos); 0.37 ± 0.19 nM (*lat-1* embryos); 1.9 ± 0.7 nM (wild-type adults); 2.3 ± 0.8 nM (*lat-1* adults). Assays were performed in three independent experiments, data are given as means ± SD. * p < 0.05; ** p < 0.01; *** p < 0.001. (I-L) Relative positions of ABala, ABalp, and MS in an AB^4^ embryo. Schematic representation (top) and DIC microscopy (bottom), scale bars = 10 μm. In wild-type embryos (I) and embryos incubated in 80 μM forskolin for 120 minutes (K) ABal divides in an anterior-posterior direction with only ABalp forming an interface with MS. In *lat-1* mutants the division plane is skewed resulting in ABala and ABalp contacting MS (J). *lat-1* embryos treated with 80 μM forskolin for 120 minutes display wild-type division and cell interfaces (L). (M) Arrangement of blastomeres in the 12-cell stage embryo. 3D representation of the data shown in Fig 2I-L using a wild-type, a *lat-1* embryo and a *lat-1* embryo treated with 80 μM forskolin (fsk) show that defects in anterior-posterior division plane alignment in *lat-1* mutant embryos are changed towards wild-type cleavage orientations upon forskolin treatment. Left-hand side shows the positions of the 8-AB descendants and the MS blastomere are shown for the individual embryos. The right-hand side is two views of the mean positions of the blastomeres. The spheres are enlarged to convey an impression of the contacts. Forskolin turns the ABal spindle in the mutant in a more anterior direction. (N) Cell division plane angles of ABal relative to MS of untreated wild-type/*lat-1* controls and wild-type/*lat-1* embryos after incubation of mothers and subsequently embryos for 48 hours in 80 μM forskolin. Upon forskolin treatment, mutant embryos display a wild-type division angle. As controls, nematodes were treated with 0.8% DMSO lacking forskolin (mock). Data are shown as average angles ± SD, *** p < 0.001; n ≥ 12. (O) Forskolin reduces the contact length of ABala to MS. Ratio of relative contact lengths of ABala to MS and ABalp to MS cells *lat-1* embryos untreated (control, n = 10) and after incubation of mothers and subsequently embryos for 48 hours in 80 μM forskolin (n = 11). In wild-type (n = 10) and forskolin-treated wild-type (n = 11) embryos ABala does not contact MS. Data were calculated from pixel analyses utilizing DIC images and are shown as means ± SD, ** p < 0.01.

In embryos homozygous for *lat-1(ok1465)*, subsequently referred to as *lat-1*, the ABal division plane was tilted towards a position almost perpendicular to the anterior-posterior axis (90.3°±17.9°, means ± SD, n = 18, p < 0.001 Fig 2B and 2E) whereas the wild-type orientation was more oblique (128.2°± 8°, means ± SD, n = 14; angles measured towards the posterior; Fig 2A and 2E).

When knocking down *gsa-1* in wild-type nematodes by feeding dsRNA to young adult hermaphrodites we observed no embryonic lethality in the offspring. However, the embryos still displayed a minor turning of the ABal spindle towards the direction typical for *lat-1* (123.5° ± 7.7°, means ± SD, n = 11; Fig 2E). It appeared that RNAi affected the progeny (F1) more drastically by rendering the adult hermaphrodites almost sterile. Only few embryos could be recovered as most hermaphrodites did not contain any embryo, some, however, one to five. In these second generation (F2) embryos, the orientation of the ABal division plane was more similar to that of *lat-1* mutants (115.3° ± 12.2°, means ± SD, n = 12; Fig 2C and 2E).

In contrast to F1 embryos, the cleavage direction of F2 embryos was significantly different from wild-type embryos (p < 0.001). Although some F2 embryos showed a cleavage angle of 87°, which is typical for *lat-1* (Fig 2E), no entire similarity to *lat-1* was reached. The fact that the RNAi effect is only pronounced in the F2 generation suggests that either inactivation of the *gsa-1* mRNA is very inefficient and/or slow or that oocytes already contain a sufficient amount of maternally contributed GSA-1 to rescue embryos in the absence of endogenous mRNA. Analyses of mRNA levels by qPCR revealed a 3- to 6-fold reduction of *gsa-1* transcript in all RNAi-treated samples compared to untreated levels (S2A Fig). Protein levels of GSA-1 are significantly reduced 36 hours after onset of RNAi treatment, albeit not fully depleted (S2B and S2C Fig) suggesting that GSA-1 protein is still available in the cells but at lower levels than in wild-type animals.

Although the cleavage direction of *gsa-1* RNAi embryos is the same as in *lat-1* mutants this is not a stringent indication that both proteins function in the same pathway, they could still function in parallel. As *lat-1*(*ok1465)* is a null mutant, one should expect that an additional inactivation of GSA-1 should not have an effect on the cleavage direction of ABal, if they act in the same pathway. However, a synergistic effect should be visible if they work in different pathways. To analyze whether LAT-1 and GSA-1 function sequentially or independently *lat-1* mutants were fed with bacteria expressing dsRNA for the *gsa-1* sequence, which yielded a cleavage direction of ABal in F2 embryos that was not significantly altered compared to *lat-1* embryos (97.7° ± 8.8°, means ± SD, n = 13, p > 0.1; Fig 2D and 2E). This is consistent with the notion that GSA-1 is the G protein downstream of LAT-1.

To investigate the physiological relevance of the LAT-1-dependent G_s_ protein-mediated cAMP signal in *C*. *elegans*, we first measured cAMP levels in embryos. Interestingly, the cAMP level in a population of *lat-1* embryos was significantly reduced (0.26 nM) compared with wild-type embryos (0.52 nM) (Fig 2F). Next, we tested if an elevation of cAMP rescues lethality of *lat-1* worms, a consequence of the tilted ABal division plane. Consistent with the maternal and zygotic requirement of LAT-1 [14] we incubated L4 larvae and subsequently developing embryos with different compounds promoting or mimicking elevation of cAMP levels: the adenylyl cyclase activator forskolin, the phosphodiesterase inhibitor 3-isobutyl-1-methylxanthine (IBMX) and the stable cAMP analogue 8-bromoadenosine 3′,5′-cyclic monophosphate (8-Br-cAMP). Treatment with any of these compounds led to an amelioration of embryonic lethality with forskolin having the strongest effect by increasing the survival rate from 33% to 73% (Fig 2G). As forskolin activates adenylyl cyclases and thus, potentially exhibits toxicity we tested various concentrations of forskolin and found that 80 μM had an optimal effect whereas higher concentrations were detrimental on *C*. *elegans*. As shown in Fig 2H, forskolin elevated cAMP in wild-type and in *lat-1* hermaphrodites. Interestingly, we did not observe any involvement of cAMP in the LAT-1 function depending exclusively on the N terminus. As loss of this function leads to reduced fertility, we investigated the effect of elevated cAMP levels on brood size of *lat-1* mutants. We did not detect any rescue of reduced brood size upon treatment with forskolin (S3A Fig), independently of time and duration of drug application (S3B Fig), suggesting that the 7TM-independent function of LAT-1 involved in fertility does not rely on a G_s_/adenylyl cyclase-mediated cAMP signal.

We next investigated if forskolin corrects the defective ABal spindle orientation observed in *lat-1* mutant embryos (95°± 7°, means ± SD, n = 12, p < 0.0005; Fig 2J and 2N) into the wild-type direction (117° ± 9°, means ± SD, n = 13; angle measured towards the posterior; Fig 2I and 2N). Upon application of 80 μM forskolin ABal spindles in *lat-1* mutants returned to the oblique position (114° ± 10°, means ± SD, n = 14, p > 0.5) (Fig 2K–2N).

As a result of faulty cleavage plane orientation in *lat-1* mutant embryos both daughters, ABalp and ABala, retain equal contact to MS after division [14] (Fig 2J) whereas in wild-type embryos only the posterior daughter ABalp remains in contact with the MS blastomere. The anterior daughter ABala is displaced to the most anterior position in the embryo (Fig 2I). The arrangement of cells at the 12-cell stage does not solely depend on the cleavage direction of ABal but also on that of ABar, which under specific circumstances may push ABala away from MS, whose position also varies in embryos. Therefore, we investigated the contact lengths of the ABala and ABalp blastomeres with MS. The ratio of the two lengths was used as a measure for cell position. In wild-type embryos ABala normally does not contact MS (ratio of 0.00 ± 0.00), whereas in *lat-1* mutant embryos it is shifted to 0.72 ± 0.03 (Fig 2O). Forskolin strongly reduced the contact of ABala to MS. Embryos in which the contact length ratio of ABala-MS to ABalp-MS was smaller than 0.45, mostly survive. However, due to potential independent roles of LAT-1 in later processes and detrimental effects of forskolin, occasionally embryos with ratios smaller than this cut-off still died. These observations suggest that suppression of lethality in *lat-1* mutants generally occurs by correcting the aberrant cleavage direction of the ABal blastomere in the mutant. It appears surprising that forskolin, which acts on all adenylyl cyclases, has such a specific effect. Initially we suspected that the drug may universally randomize spindle orientation and thus, ABala occasionally would not touch MS. However, the standard deviation for the angle of ABal cleavage is ± 9° in untreated wild-type embryos and ± 10.2° after forskolin treatment, indicating that the drug does not randomize cleavage directions. Thus, the suppression of the *lat-1* phenotype by forskolin is very specific, suggesting that by activating adenylyl cyclases and subsequently raising cellular cAMP concentrations, it specifically mimics the process normally controlled by LAT-1.

Further, cAMP being the central element in this process is also supported by the fact that decreasing its levels by treating very early wild-type embryos with the adenylyl cyclase inhibitor 2',5'-dideoxyadenosine (ddA) leads to a specific partial phenocopy of *lat-1* mutants resulting in embryonic lethality (S4 Fig).

Taken together, these results indicate that LAT-1 implements signaling via cAMP *in vivo*. As the *lat-1* phenotype is rescued by elevation of cAMP which is not restricted to a certain cellular compartment the division plane orientation in an anterior-posterior direction is likely to be molecularly realized by components downstream of this second messenger.

### LAT-1 functions in embryonic development upon activation by a tethered peptide agonist

**Fig 3:**
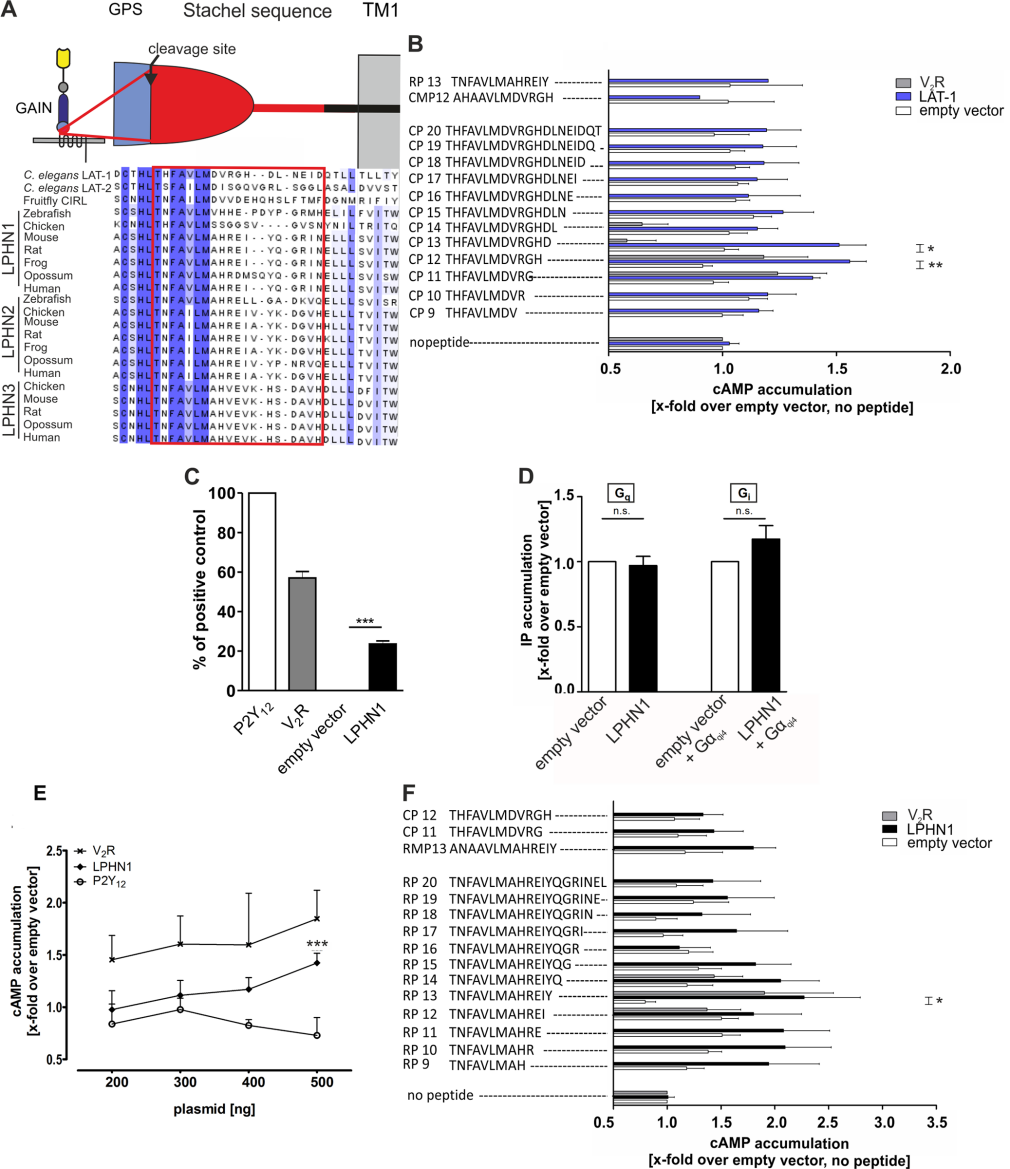
An agonistic sequence C-terminal of the GPS cleavage site activates LAT-1 *in vitro*. (A) Evolutionary conservation of the putative agonistic region C-terminal of the GPCR proteolytic site (GPS) cleavage site in different latrophilin homologs. The GPS is part of the GPCR-autoproteolysis inducing (GAIN) domain, a characteristic feature of most aGPCRs which is located N-terminal of the first transmembrane domain (TM1). Sequences were retrieved from NCBI/GenBank and aligned utilizing Jalview 2 [36]. (B, F) Peptide-stimulated cAMP response of LAT-1 (B) and rat LPHN1 (F). COS-7 cells transfected with 0.2 μg empty control vector (pcDps) or plasmid encoding the latrophilin homolog, respectively, were stimulated with 1 mM peptide and cAMP levels were measured by cAMP accumulation assay. A mutated peptide served as negative control (CMP12/RMP13). As control for peptide specificity, the human vasopressin type 2 receptor (V_2_R) was used, which does not respond to any of the peptides tested. Basal cAMP levels (empty vector, no peptide) are 5.3 ± 1.3 nM. Data are given as means ± SD of five independent experiments, each performed in triplicate. * p < 0.05; ** p < 0.01. (C) LPHN1 is expressed in COS-7 cells. Cells were transfected with 500 ng/well of empty vector (pcDps) or plasmid encoding either rat LPHN1, human P2Y_12_, or human V_2_R. After 48 hours, surface expression levels were determined using surface ELISA and displayed as percentage of positive control P2Y_12_. Expression of the human vasopressin type 2 receptor V_2_R is shown as a comparison. The non-specific OD value (empty vector) was 0.06 ± 0.03 (set 0%) and the OD value of P2Y_12_ was 1.28 ± 0.12 (set 100%). Data are given as means ± SD of at least three independent experiments. *** p < 0.001. (D) LPHN1 does not yield a basal signal for Gα_i_- or Gα_q_-protein coupling. Testing G_i_ and G_q_ protein-signal specificity of rat LPHN1, IP accumulation assays were performed to detect G_q_ protein-mediated increase in IP levels. To analyze functional G_i_ coupling, the chimeric Gα_qi4_ protein was applied to reroute a potential G_i_-protein pathway to the G_i_ protein-mediated signaling cascade. For each assay, 1.5 μg/well of plasmid encoding LPHN1 was transfected (for G_i_-protein coupling, co-transfection with 100 ng of chimeric Gα_qi4_ protein was performed). No activation of any of the signaling pathway was observed. Basal IP levels are: 220 ± 34 CPM/well (empty vector); 234 ± 39 CPM/well (empty vector + Gα_qi4_). Data are given as means ± SD of three independent experiments, each performed in triplicate. n.s. not significant. (E) LPHN1 couples to Gα_s_. COS-7 cells were transfected with increasing amounts of empty control vector (pcDps) or plasmid encoding either rat LPHN1, human V_2_R or human P2Y_12_, and cAMP levels were measured after 48 hours. cAMP concentrations are shown as fold change over empty control vector, basal cAMP levels: 6.2 ± 2.3 nM (200 ng); 6.1 ± 2.5 nM (300 ng); 5.1 ± 2.6 nM (400 ng); 6.1 ± 4.5 nM (500 ng). The G_s_-protein coupling V_2_R served as positive and the predominantly G_i_-protein coupling P2Y_12_ as negative control. Data are given as means ± SD of three independent experiments, each performed in triplicate. *** p < 0.001.

We next asked how LAT-1 is activated to trigger the signaling cascade to ensure correct cell division plane orientation. Previously, we have postulated that an interaction between the GPS and 7TM domain is essential for LAT-1 activation [25]. Very recently, Liebscher *et al*. have shown for the aGPCR GPR126 in zebrafish that the sequence immediately C-terminal of the cleavage site in the GPS (the *Stachel* sequence) acts as an internal tethered agonist to activate GPR126 [35]. We hypothesized that in LAT-1 a similar sequence would also have agonistic properties. To test this hypothesis, we analyzed peptides of varying lengths comprising the sequence C-terminal of the GPS cleavage site (Fig 3A) for their ability to activate LAT-1 *in vitro* by measuring cAMP levels. Peptides of 12 and 13 amino acids (CP12, CP13) increased cAMP levels in *lat-1*-transfected COS-7 cells above basal levels, with CP12 displaying the highest agonistic activity by increasing the concentration 1.6-fold (Fig 3B). In contrast, a CP12-derived peptide with positions +1 (T530 in the full length receptor) and +3 (F532 in the full length receptor) mutated to alanine did not display any activity (CMP12) demonstrating that agonistic activity is highly sequence-specific (Fig 3B). The agonistic peptides were able to activate a chimeric LAT-1 with the extracellular N terminus exchanged for that of the rat muscarinic M_3_ acetylcholine receptor (M3R ECD::LAT-1, S5 Fig) suggesting that the sequence directly interacts with the 7TM.

As our initial experiments tested G protein-coupling abilities of LAT-1 only under non-stimulated conditions (Fig 1C) we performed these assays in the presence of the agonistic peptide CP12 to elucidate coupling to G_i_ and G_q_ proteins. However, we were unable to obtain any results with these chimeric G proteins. Due to rather low LAT-1 expression levels compared to mammalian G_i_ or G_q_-coupled receptors we cannot fully exclude the possibility of coupling to other G protein families additionally to G_s_ proteins.

We next tested whether activation by a tethered agonist is conserved between species. In mammals, three latrophilin homologs exist. LPHN1 (ADGRL1) shows the highest conservation to LAT-1 overall and within the potential agonistic sequence (Fig 3A). Like LAT-1, this receptor is expressed in COS-7 cells (Fig 3C) and slightly activates the G_s_-signaling pathway, but not a G_q_- or G_i_-signaling cascade in the absence of an agonist (Fig 3D and 3E). Further analyses showed that, similar to *C*. *elegans* LAT-1, rat LPHN1 was activated by a peptide representing the sequence C-terminal of the cleavage site. A peptide of 13 amino acids length (RP13) displayed the most efficient agonistic properties and increased basal cAMP levels 2.3-fold (Fig 3F). However, we did not observe any cross-activation of LAT-1 with the rat *Stachel* sequence and *vice versa* (Fig 3B and 3F). We also tested if the agonistic peptides are able to trigger a G_q_ or G_i_ pathway via rat LPHN1. Upon stimulation with the agonistic peptide RP13 coupling of the receptor to a G_q_ protein was observed in an IP accumulation assay (S6 Fig). These data suggest that the activation mechanism involving the *Stachel* sequence is conserved between species, but the implementation of the mechanism is sequence-specific, at least between distantly related latrophilin orthologs.

**Fig 4:**
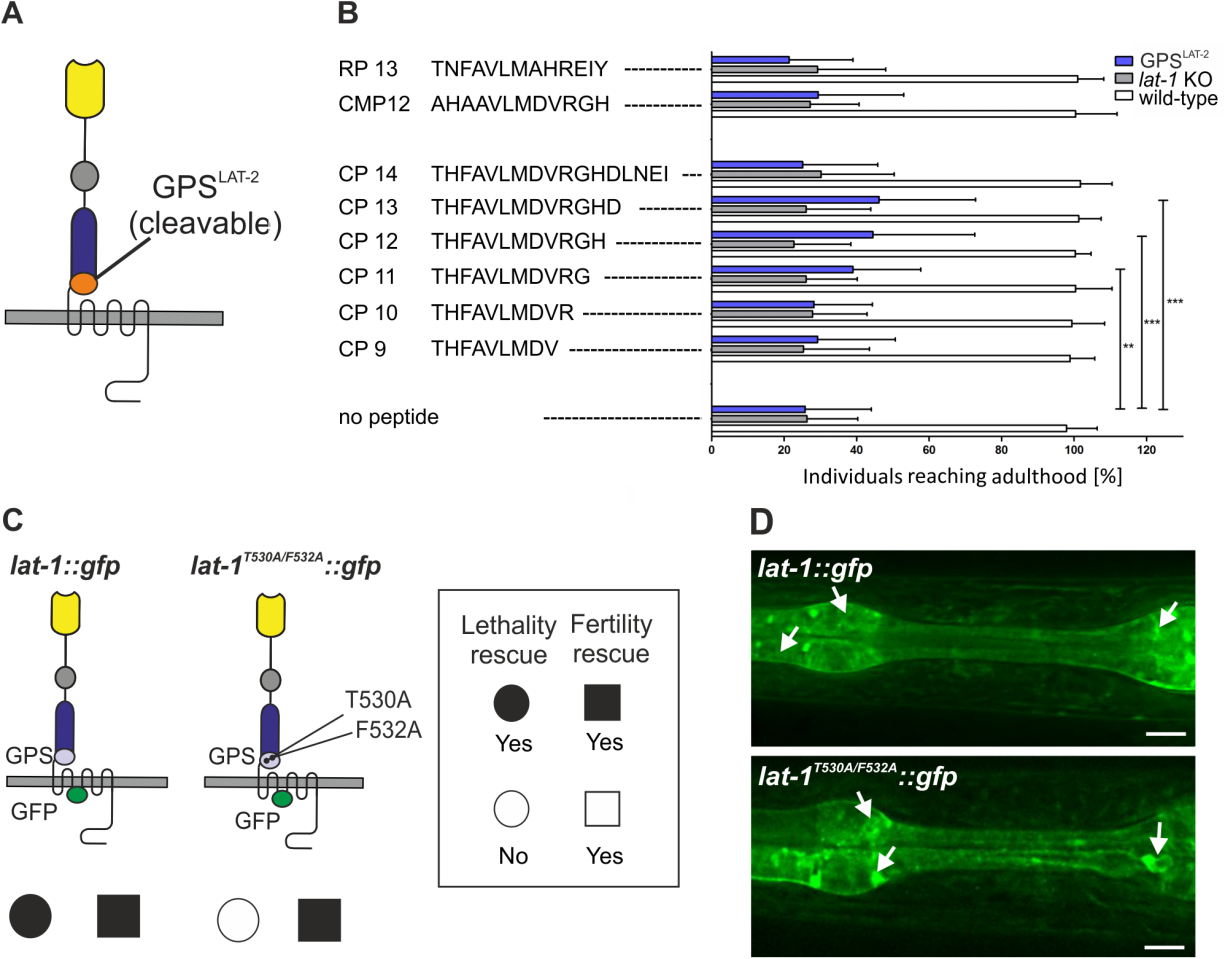
The agonistic sequence of LAT-1 triggers receptor function *in vivo*. (A) Domain architecture of LAT-1 with an exchange of the GPS for the LAT-2 GPS. (B) Peptide-mediated rescue of lethality in *lat-1* mutant nematodes expressing *lat-1* with a *lat-2* GPS. Mothers and subsequently offspring were soaked in 0.1 mM of peptide and individuals reaching adulthood were scored (n ≥ 350). No response to any peptide was observed in *lat-1* null mutant animals. Negative controls: mutated peptide CMP12 and a peptide derived from rat LPHN1 (RP13). Data are shown as means ± SD, **p<0.01; ***p<0.001. (C) Transgenically expressed *lat-1* ^*T530A/F532A*^::*gfp* exclusively rescues fertility defects of *lat-1* mutants. Nematodes expressing *lat-1* ^*T530A/F532A*^::*gfp* in a *lat-1* mutant background display lethality but no fertility defects compared to a *lat-1*::*gfp* transgenic control. (D) Expression and protein localization of *lat-1*::*gfp* (top) is indistinguishable from *lat-1* ^*T530A/F532A*^::*gfp* (bottom). Arrows indicate expression.

To assess the agonistic properties of the *Stachel* sequence *in vivo* and its impact on LAT-1 signaling in embryonic development we employed rescue experiments utilizing *lat-1* mutant animals expressing a chimeric receptor. In this receptor the LAT-1 GPS and thus, the entire *Stachel* sequence is exchanged for the GPS of *C*. *elegans* LAT-2 (Fig 4A). This receptor has been shown to retain activity to complement the fertilization defect in *lat-1* mutants, but does not rescue the tissue polarity phenotype [25], ensuring that it is devoid of any potential activation by the *Stachel* sequence. In this assay, soaking of hermaphrodites with the agonistic peptides CP11, CP12 and CP13 efficiently rescued the tissue polarity phenotype in the early embryo, demonstrating that all three peptides are also able to activate LAT-1 *in vivo* (Fig 4B). CP13 had the strongest effect (46 ± 12%) compared to untreated controls (26 ± 7%). Peptides with no agonistic activity *in vitro* (CP9, CP10, CP14, RP13, CMP12) did also not display any activity *in vivo* (Fig 4B). To test whether this effect is specific to LAT-1 signaling, we also treated *lat-1* mutants with the respective peptides but did not detect any rescue (Fig 4B). We did not observe any effect of these peptides on wild-type nematodes, which might be due to the fact that receptor signaling is tightly controlled *in vivo* or that an increase in activity levels is not reflected in a specific phenotype. To further control for specificity we introduced mutations in the *Stachel* sequence of the full length LAT-1 protein (T530A and F532A, similarly to the mutations described in CMP12). Consistent with the *in vitro* experiments no rescue of developmental lethality was detected when expressing this construct in *lat-1* mutant nematodes (Fig 4C). To ensure that the mutant protein is functional we assessed the expression of the *lat-1* ^*T530A/F532A*^::*gfp* construct, which is indistinguishable from a wild-type receptor (Fig 4D). Biochemical activity was confirmed by the rescue of the reduced fertility in *lat-1* mutants, therefore confirming the receptor´s 7TM-independent functionality (Fig 4D) [25]. These data show that the sequence immediately C-terminal of the GPS cleavage site in LAT-1 is an agonistic region essential for receptor activation and thus, crucial for LAT-1 signaling in embryonic development.

## Discussion

Oriented cell division and spindle orientation in early *C*. *elegans* embryogenesis are controlled by complex signaling pathways involving GPCRs such as the four frizzled family Wnt receptors [9,37–39] and in which the role for heterotrimeric G proteins has been firmly demonstrated in several elegant studies [40,41]. Gotta and Ahringer showed that proper spindle directionality of the cleavages in the 4-cell *C*. *elegans* embryo depends on Gß/γ and that Gα signaling is required for spindle placement in the 1-cell embryo [41]. However, there is evidence that the activity of the G proteins is modulated mostly in a GPCR-independent manner via G-protein regulators [42] and GEF proteins [43]. A GPCR-dependent and G protein-mediated signaling pathway has not been unambiguously defined despite some clear indications [44,45]. In the present study, we demonstrate that a GPCR-dependent G protein-mediated signal based on the adhesion GPCR LAT-1, which is involved in orienting spindles in an anterior-posterior direction in ABal descendants [14], is an essential mechanism for controlling oriented cell division. We provide functional evidence that LAT-1 couples to a Gα_s_ protein which activates an adenylyl cyclase, thereby elevating cellular concentrations of the second messenger cAMP *in vitro* (Figs 1B and 3B). Similar coupling abilities were observed for the latrophilin ortholog rat LPHN1 (Fig 3E and 3F). consistent with analyses showing that this receptor triggers elevated cAMP levels upon treatment with α-latrotoxin *in vitro* [46]. In accordance with this study we also observe that LAT-1 triggers a change in basal IP levels, albeit only upon stimulation with an agonistic peptide (S6B Fig).

*In vivo* analyses have revealed that the increase of cAMP levels is a key signal for the anterior-posterior orientation of cleavage planes in the ABal cell in the *C*. *elegans* embryo. Treatment of *lat-1* mutant nematodes with the adenylyl cyclase activator forskolin raises cAMP independent of the receptor to a point where cell division plane orientation is sufficiently restored and subsequent lethality is rescued (Fig 2H–2O). The small contact lengths between ABala and MS cells observed in some cases does not seem to have a detrimental effect on developing embryos. The incomplete reduction of contacts lengths to wild-type levels might be due to limitations in accessibility of forskolin to the embryo. Limited drug uptake may explain the partial rescue of lethality by the different compounds affecting cAMP levels (Fig 2G). However, it is also possible that lethality cannot be rescued to wild-type levels as it might be a result of different LAT-1 functions. As neither drug accessibility nor uptake rate or half-life of each compound in the organism could be resolved, we were unable to determine the exact time point and duration at which the signal is required. However, consistent with previous work demonstrating maternal and zygotic requirement of the receptor [14], treatment of L4 hermaphrodites and subsequently embryos with the respective drug was sufficient for rescue (Fig 2G).

Consistently with the putative role of LAT-1 as a regulator of cAMP, *lat-1* mutant embryos display decreased levels of the second messenger. We cannot exclude that this decrease is exclusively a consequence of absence of LAT-1 signals in the cells of the early embryo. As there is evidence for the receptor to mediate effects in other cells as well [14], it is conceivable that these are also mediated via cAMP.

Combined with the functional *in vitro* data our analyses on rescue of *lat-1* mutant defects by stimulating a G_s_-mediated signal suggest that a GPCR cascade via a cAMP regulation is one essential pathway for the coordination of anterior-posterior cell division plane orientation in embryogenesis.

Our data indicate that the G protein involved in this cascade is GSA-1 (Fig 2A–2E). The major pathways involved in polarity decisions in the early embryo such as Notch/Delta and Wnt/Fz have been shown to mediate signals via routes different from G proteins [4,13]. However, the Wnt/Fz pathway component APC in *C*. *elegans* feeds into Rac [45] which supports a scenario in which spindle directions are also regulated by differential G-protein signaling. The signals mediated by these G proteins remain widely elusive, but involvement of different intracellular signals warrants a precise regulation and avoids intersection of the multitude of signaling pathways required to ensure tightly controlled oriented cell division. It could be speculated that LAT-1 contributes an additional signal via a G_s_ protein/adenylyl cyclase introducing a new level of regulation.

Interestingly, our data suggest that the cAMP-based signal mediating this polarity is not polar and thus, LAT-1 is not required to be asymmetrically localized prior to ABal cell division. Consistently, no asymmetrical distribution of LAT-1 has been found previously [14]. This is in contrast to asymmetric protein localization that has been described for many components of pathways involved in planar cell polarity (PCP) or Wnt/Fz signaling [3] but in accordance with some anterior-posterior tissue polarity models [47]. However, it is well possible that the signal transduced by LAT-1 to mediate an effect on polarity is polarized further downstream through effectors of cAMP such as protein kinases A (PKA) or A kinase anchoring proteins (AKAP). This effect has been shown for a cAMP-dependent PKA in the establishment of neuronal polarity [48,49]. It is also conceivable that the polarized effect of LAT-1 signaling is promoted by a temporal cue which could not be investigated in this study.

In order to transduce signals LAT-1 is activated by a tethered agonist downstream of the GPS. *In vitro* and *in vivo* analyses identified a sequence of 12 amino acids exhibiting agonistic properties (Figs 3B and 4B). These findings are in accordance with recent data on GPR126 and GPR133 which revealed similar agonistic regions, termed *Stachel* sequences [24]. The biological implications of both studies are intriguing as they raise the hypothesis that several aGPCR share the same mechanism of activation.

Our data suggest that the activation mechanism is evolutionary conserved as latrophilin orthologs in rat and *C*. *elegans* both display an intrinsic agonistic sequence of a similar lengths. However, the tethered agonist of one latrophilin receptor is highly sequence-specific (Fig 3F), it is not able to activate its ortholog despite 59% identity/76% similarity of both agonistic motifs. As mutations at the conserved positions +1 and +3 within the *Stachel* sequence are sufficient to abolish LAT-1 function this specificity is likely to be conferred by structural properties. Consistently, an exchange of the GPS for the one of the paralog LAT-2 (69% identity/87% similarity within the *Stachel* sequence) results in a loss of LAT-1 function in development [25]. These data are also in concordance with the study identifying the *Stachel* sequence of GPR126 in which no cross-activation of *Stachel* sequence-derived agonistic peptides from GPR126 and another aGPCR, GPR133, is observed despite a high amino acid identity among the agonistic regions [24]. However, cross-activation between certain aGPCR orthologs or closely related aGPCRs via the *Stachel* sequence cannot be excluded and might have interesting implications.

Interestingly, the *Stachel* sequence identified in both latrophilin orthologs corresponds exactly to the respective C-terminal section of the GPS. These findings support our previous structure-function analyses which provided evidence for an interaction between LAT-1 GPS and 7TM domain [25]. Future studies need to focus on the details of the activation mechanism and clarify how the interaction is induced as well as which region of the 7TM is a potential interaction site. As we have previously shown that cleavage at the GPS is not essential for LAT-1 function, a model in which the tethered agonist functions after receptor cleavage is unlikely. Conformational changes within the GPS-containing GAIN domain upon binding to extracellular proteins could be the stimulus for exposure of the sequence within the GPS. The 7TM-independent function of LAT-1 is not based on a cAMP signal. However, the separation of this function from the 7TM-dependent function is likely to be conferred by the tethered agonist.

**Fig 5:**
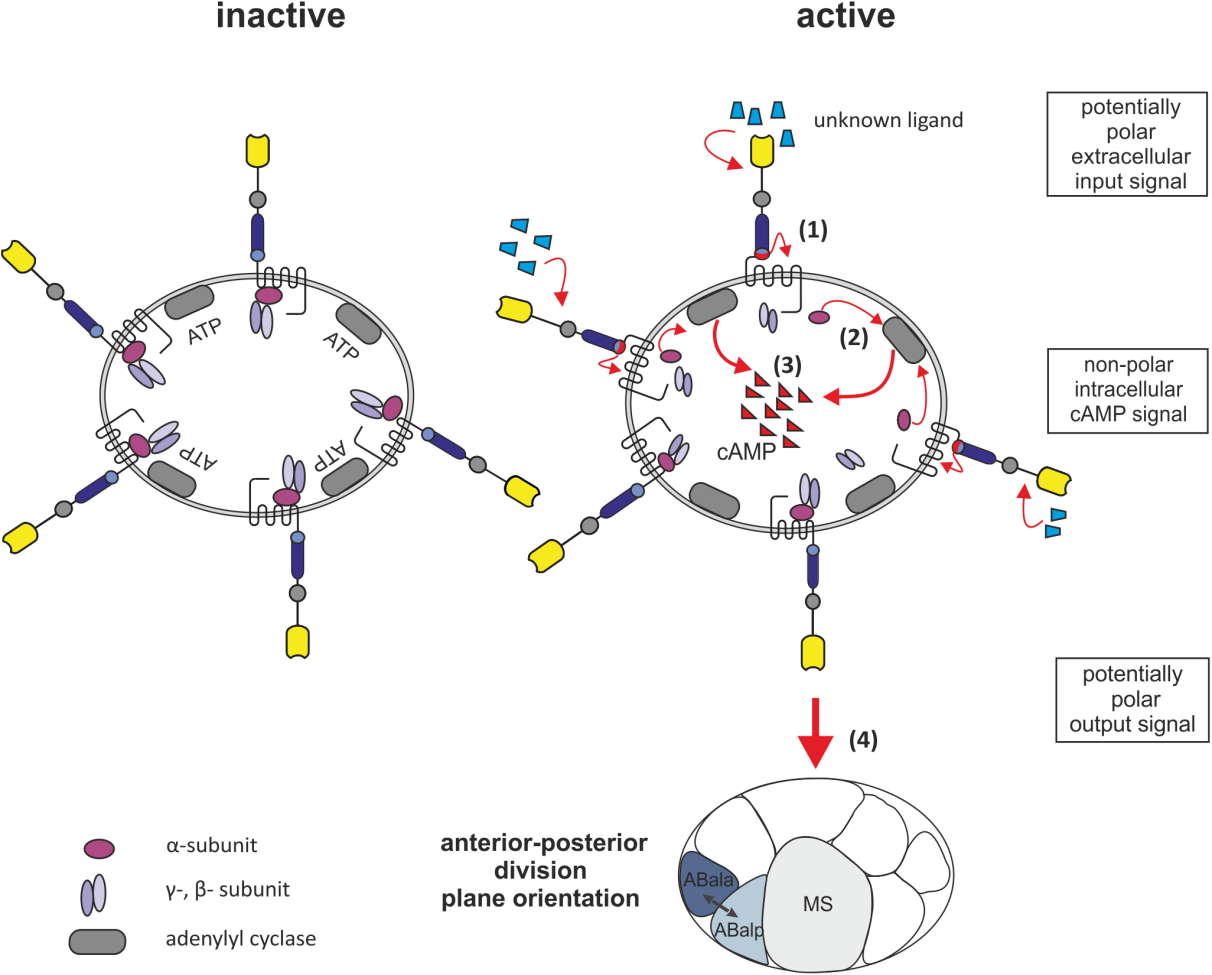
Model for LAT-1 signaling in the early embryo. LAT-1 resides in an inactive state while the *Stachel* sequence is not interacting with the 7TM domain. Upon structural changes of the N terminus e.g. binding of an as yet unknown extracellular ligand, the tethered agonist contacts the 7TM domain (1). The G_s_ protein, likely to be GSA-1, then activates the adenylyl cyclase (2) resulting in an increase of cAMP levels (3). This signal then promotes coordination of anterior-posterior cleavage plane orientation after the fourth round of cell division (4). The cAMP signal within the cell is non-polar.

In summary, our results show that a GPCR-dependent G protein-signaling cascade based on LAT-1 is involved in oriented cell division in the early *C*. *elegans* embryo. LAT-1 activates a G_s_ protein/adenylyl cyclase signaling pathway, probably via GSA-1. By regulating cAMP levels, the receptor controls coordination of anterior-posterior cleavage plane orientation in the ABal cell. The data support a model in which LAT-1 resides in an inactive state while the *Stachel* sequence is not interacting with the 7TM domain (Fig 5). We hypothesize that an unknown extracellular cue causes the tethered agonist to contact the 7TM domain resulting in an increase of cAMP levels. This signal then promotes coordination of anterior-posterior cleavage plane orientation after the fourth round of cell divisions. Future studies need to focus on the effectors of the cAMP signal and how polar division plane orientation is coordinated on a molecular level by the identified non-polar signal.

## Materials and Methods

### *C*. *elegans* strains

*C*. *elegans* strains were cultured and manipulated according to standard protocols [50]. Wild-type worms were *C*. *elegans* variety Bristol, N2. The allele *lat-1(ok1465)* was generated by the *C*. *elegans* gene knockout consortium and provided by the Caenorhabditis Genetics Center (CGC), which is funded by NIH Office of Research Infrastructure Programs (P40 OD010440). The following transgenes have been previously described: *aprEx157[lat-1(lat-2 GPS) (pSP75) rol-6(su1006) pBSK]* [25], *aprEx77[lat-1*::*gfp (pSP5) rol-6(su1006) pBSK]* [14]. The transgene *aprEx185[lat-1* ^*T530A/F532A*^ *(pSP94) rol-6(su1006) pBSK]* was generated for this study (for details see Supporting Experimental Procedures).

### *In vitro* functional assays

For functional assays, COS-7 cells were transiently transfected. To determine total as well as cell surface expression of receptors carrying N-terminal HA and C-terminal FLAG tags, indirect cellular enzyme-linked immunosorbent assays (ELISA) were employed [51]. cAMP concentrations were measured using the ALPHAScreen cAMP assay kit (PerkinElmer Life Sciences) according to the manufacturer's protocol. IP formation was determined as previously described [31]. Assay data was analyzed with GraphPad Prism version 5.0 (GraphPad Software). Statistics were performed using a two-way ANOVA in combination with Bonferroni as post-hoc test. For details see Supporting Experimental Procedures.

### Drug and peptide experiments

All compounds were obtained from Sigma Aldrich. Forskolin was dissolved in DMSO to 10 mM, 3-isobutyl-1-methylxanthine (IBMX) in Dent´s buffer to 10 mM, 8-bromoadenosine 3′,5′-cyclic monophosphate (8-Br-cAMP) in 1 N ammonium hydroxide to 250 mM and 2',5'-dideoxyadenosine (ddA) was diluted in DMSO to 100 mM prior to final dilution in Dent´s buffer. Peptides were synthesized (see Supporting Experimental Procedures) and dissolved in DMSO to 100 mM prior to final dilution in M9. Worms were incubated in 80 μM forskolin, 10 mM IBMX, 0.25 mM 8-Br-cAMP, 1 mM ddA or 0.1 mM peptide solution, respectively, containing *E*. *coli* OP50.

### RNA interference

RNAi of *gsa-1* was carried out using a feeding clone. The open reading frame of *gsa-1* was amplified from total cDNA using primers RNAi_1/RNAi_2 (for primer sequences see S1 Table) and cloned into pCR4 (Life Technologies). It was then cloned into the *Not*I/*Spe*I sites of L4440 [52]. Feeding by RNAi was performed as previously described using the *E*. *coli* strain HT115 [53]. Embryos of the F1 and the F2 generation fed with the RNAi clone described were analyzed for spindle orientation in dividing blastomeres using 4D microscopy.

### Quantitative PCR (qPCR)

Nematodes were collected and approximately 2,000 hermaphrodites were placed in TRIzol (Thermo Fisher Scientific). Total RNA was extracted following the manufacturer´s protocol. cDNA was obtained from 1 μg RNA using Omniscript RT kit (Qiagen) and random hexamer primers. qPCR analysis of *gsa-1* was performed with primers lat1_1034F/lat1_1035R using a LightCycler PCR machine and GoTaq® qPCR Master Mix (Promega) according to manufacturer´s protocol. As internal reference genes the following were used: *act-1* (primers SP1/SP2), *cdc-42* (primers SP3/SP4), *eif-3* (primers SP7/SP8), *tba-1* (primers SP9/SP10). For primer sequences see S1 Table. Data analysis was performed utilizing MxPro QPCR Software (Agilent Technologies).

### Western blot analysis

Approximately 2,000 hermaphrodites were placed in 100 μl M9 containing protease inhibitor (Roche) and sonicated with 15 30 s pulses in a Bioruptor Standard (Diagenode). Approximately 20 μl of sample were boiled in Laemmli buffer for 5 minutes. For the mammalian cell control, 3 × 10^5^ HEK293 cells were lysed in Laemmli buffer for 5 minutes. Protein was subject to electrophoresis as described previously [54] using a 12.5% sodium dodecyl sulfate-polyacrylamide gel electrophoresis (SDS-PAGE) gel and transferred to a nitrocellulose membrane (Amersham). Blots were probed with rabbit anti-Gsα antibody (Merck Millipore) at 1:1,000 dilution overnight at 4°C and subsequently incubated for 2 hours at room temperature with a horseradish-peroxidase-conjugated goat anti-rabbit antibody (Sigma Aldrich) at 1:10,000 dilution. Western blots were developed by an enhanced chemiluminescence (ECL) detection system (Thermo Fisher Scientific). For detection of actin as loading control, membranes were stripped in Stripping buffer (1% SDS, 0.1 M Tris pH 6.8, 0.175% β-mercaptoethanol) for 30 minutes at 50°C, blocked and probed with mouse anti-actin (Merck Millipore) at 1:1,000 dilution, and then incubated with horseradish-peroxidase-conjugated rabbit anti-mouse (Sigma Aldrich) 1:10,000 and processed as described above. Antibody signals were quantified by densitometric analysis using ImageJ software [55].

### Microscopy

4D DIC imaging and quantitative evaluation of division plane angles were performed as previously described using SIMI Biocell software (SIMI Reality Motion Systems) [56]. Embryos were dissected from young adult hermaphrodites incubated for 120 minutes in Dent`s buffer, 80 μM forskolin or respective solvents as control. Live images were taken with a Zeiss Axioplan 2e and a Zeiss Examiner. Z-stacks with spatial spacing of 1 μm were taken every 35 ms for 300 min. Confocal and fluorescent images were collected with Zeiss LSM5 and LSM510 Meta setups.

### Lethality rescue assay

The lethality rescue assay was conducted as previously described [14]. Fifty L4 hermaphrodites were transferred into wells of a 72-well flat-bottom Terasaki plates (Greiner Bio-One) containing OP50 and allowed to lay eggs for 24 hours at 22°C. Five to ten eggs were transferred into fresh wells with corresponding solutions and incubated at 22°C. The number of dead/surviving embryos was scored 24 hours later, the number of adult animals 48 hours later on an inverted microscope. Data were examined with an unpaired two-tailed t test for each genotype and condition.

## Supporting Information

S1 Text More detailed description of material and methods. (DOCX)

S1 Fig Total expression of *lat-1* in COS-7 cells. COS-7 cells were transfected with 1 μg of empty vector (pcDps) or plasmid encoding either human ADP receptor P2Y_12_, the human vasopressin type 2 receptor V_2_R or LAT-1. Total expression levels were measured 48 hours post transfection using ELISA. Data are displayed as percentage of P2Y_12_ (positive control) and given as means ± SD of five independent experiments, each performed in triplicate. The non-specific OD value (empty vector) is 0.06 ± 0.01 (set 0%) and the OD value of P2Y_12_ is 0.92 ± 0.02 (set 100%). *** p < 0.001.
(TIF)

S2 Fig RNAi reduces transcript and protein levels of *gsa-1*. (A) qPCR analysis of *gsa-1* in adult wild-type hermaphrodites after 24 hours and 36 hours *gsa-1* RNAi and in adult F1 hermaphrodites. Already after 24 hours a knockdown of *gsa-1* transcript levels are detected compared to nematodes fed with empty vector (L4440). A complete knockdown is not achieved. Data are normalized using the geometric mean of the reference genes *act-1*, *cdc-42*, *eif-3* and *tba-1* and shown as mean ± SD of three independent experiments each performed in triplicate. (B, C) GSA-1 levels in wild-type hermaphrodites after 24 hours and 36 hours *gsa-1* RNAi. Western blot analyses using an anti-Gα_s_ antibody show an increasing reduction of protein with time. The antibody recognizes an epitope sequence which is conserved between mammals and *C*. *elegans* and thus, probes mammalian Gα_s_ as well as nematode GSA-1 (B), which are both 46 kDa. HEK293 cells served as mammalian cell control. Actin was used as a loading control (42 kDa). To examine the level of GSA-1 reduction, densitometric analyses of the Western blots were performed (C). Pixels of the different bands and respective backgrounds were quantified and data normalized to the pixel intensity of the respective actin band. Data are given as mean ± SD of three independent experimental analyses. Note that protein of F1 *lat-1; gsa-1(RNAi)* nematodes was not analyzed due to too little quantities of respective nematodes.
(TIF)

S3 Fig A cAMP-mediated signal is involved in the role of LAT-1 in cleavage plane orientation but not in fertility. (A) Fertility defects of *lat-1* mutants are not rescued by treatment of hermaphrodites from embryonic stages to adulthood with 80 μM forskolin. Brood size was scored compared to untreated (mock), DMSO-treated (0.8%) and wild-type control. Data are given as means ± SD, n.s. not significant. (B) Forskolin treatment (80 μM) does not rescue fertility defects in *lat-1* mutants independently of the time point of drug application. Rescue of developmental lethality in *lat-1* mutants occurs only when adding forskolin to the mother or the very early embryonic stages.
(TIF)

S4 Fig Inhibition of adenylyl cyclases in wild-type hermaphrodites partially mimics the *lat-1* phenotype. Treatment of adult hermaphrodites and subsequently very early wild-type embryos with the adenylyl cyclase inhibitor 2',5'-dideoxyadenosine (ddA) leads to specific embryonic lethality only observed in wild-type individuals but not in *lat-1* mutant embryos. Note that this effect might be not fully penetrant due potential difficulties in drug accessibility. As controls, nematodes were treated with 1% DMSO lacking ddA (mock). Individuals surviving embryogenesis were scored (n > 520). Data are given as means ± SD. n.s. not significant; *** p < 0.001.
(TIF)

S5 Fig Agonistic peptides activate a chimeric LAT-1 with the extracellular N terminus of rat M3R (M3R ECD::LAT-1). Peptide-stimulated cAMP response of M3R ECD::LAT-1. COS-7 cells transfected with 200 ng empty control vector (pcDps) or plasmid encoding M3R ECD::LAT-1 were stimulated with 1 mM peptide. Subsequently, cAMP levels were measured by cAMP accumulation assay. The human vasopressin type 2 receptor (V_2_R) served as control for peptide specificity, which does not respond to any of the peptides tested. Basal cAMP levels (empty vector, no peptide) are 5.3 ± 1.3 nM. Data are given as means ± SD of five independent experiments, each performed in triplicate. * p < 0.05; ** p < 0.01.
(TIF)

S6 Fig LPHN1 signals via coupling to a G_q_ protein but not a G_i_ protein upon activation by an agonistic peptide. (A) Total levels of LPHN1 in COS-7 cells determined by ELISA. Expression levels were measured 48 hours after transfection with 1 μg of empty vector (pcDps) or plasmid encoding either human ADP receptor P2Y_12_, the human vasopressin type 2 receptor V_2_R or rat LPHN1. Data are displayed as percentage of P2Y_12_ (positive control) and given as means ± SD of five independent experiments, each performed in triplicate. The non-specific OD value (empty vector) is 0.06 ± 0.01 (set 0%) and the OD value of P2Y_12_ is 0.92 ± 0.02 (set 100%). (B) COS-7 cells transfected with 1.5 μg empty control vector (pcDps) or plasmid encoding LAT-1 were stimulated with 1 mM peptide and IP accumulation assays were performed. An increase in IP levels upon stimulation with peptide RP12 indicates coupling of LPHN1 to a G_q_ protein. RMP12 served as negative control peptide. Basal IP levels (empty vector, no peptide) are 452 ± 101 CPM/well. Data are normalized to respective non-stimulated controls and are given as means ± SD of two independent experiments, each performed in triplicate. n.s. not significant; * p < 0.05.
(TIF)

S1 Table Sequence of primers used for qPCR analyses and to generate constructs presented in the study. (XLSX)
