# Supplementary material for: Oriented Cell Division in the C. elegans Embryo Is Coordinated by G-Protein Signaling Dependent on the Adhesion GPCR LAT-1
Source: PLoS Genet. 2015 Oct 27;11(10):e1005624. doi: 10.1371/journal.pgen.1005624 (PMC4624771; doi:10.1371/journal.pgen.1005624)
Supplement: S1 Text — (DOCX) [file pgen.1005624.s001.docx]

**S1 Text. More detailed description of material and methods**

**Generation of plasmids and transgenes**

Constructs for *in vitro* functional assays were generated by cloning cDNA into the mammalian expression vector pcDps. For generation of constructs for *in vivo* analyses recombineering was employed [[1](#_ENREF_1),[2](#_ENREF_2)] and accompanying protocols were modified as previously described [[3](#_ENREF_3),[4](#_ENREF_4)] to construct latrophilin transgenes using cosmids, PCR-amplified targeting cassettes and positive antibiotic selection.

*lat-1* in pcDps (pSP101)

The sequence of *lat-1* without the predicted signal peptide [[5](#_ENREF_5)] was amplified from a *C. elegans* cDNA library with primers lat1_604F/ lat1_445R (for primer sequences see S1 Table). The predicted signal peptide sequence of *lphn1* was amplified from a mouse brain cDNA library with primers lat1_602F/lat1_603R. An N-terminal hemagglutinin (HA) epitope tag and a C-terminally FLAG epitope tag were inserted via the primers. Both fragments were phosphorylated, ligated together and digested with *Kpn*I and *Eco*RI to generate compatible ends for ligation into pcDps digested with the same enzymes.

*M3R ECD::lat-1* in pcDps (pAM11)

The sequence of the extracellular N terminus of the muscarinic M3 receptor (M3R) was amplified from a mouse brain cDNA library with phosphorylated primers lat1_707F/lat1_708R (for primer sequences see S1 Table).

To replace the sequence of the extracellular domain of *lat-1*, pSP101 including murine *lphn1* signal peptide sequence, N-terminally HA epitope and C-terminally FLAG epitope was amplified with primers lat1_709F/lat1_710R. The PCR fragment was digested with *Dpn*I and ligated with the sequence of the M3R N terminus.

Rat *lphn1* in pcDps (pSP113)

The sequence of rat *lphn1* cDNA was amplified from LPH42 (kindly provided by Y. Ushkaryov) with primers lat1_890F/lat1_891R and phosphorylated. pcDps vector backbone containing HA epitope tag and FLAG epitope tag was amplified with primers lat1_863F/lat1_864R from pSP101, digested with *Dpn*I and ligated with the rat *lphn1* cDNA fragment.

*lat-1^T530A/F532A^* (pSP94)

To generate the point mutations for pSP94, a recombineering targeting cassette consisting of two parts was generated. A 0.2 kb fragment of exon 5 was amplified from pSP5 [[3](#_ENREF_3)] with forward primers introducing an ACA to GCA change for T530A and a TTT to GCA change for F532A (primers rec_144F/rec_145R). The reverse primer contained an overhanging *Hin*dIII site. The second part of the cassette was the FRT-kanR-FRT cassette amplified from pIGCN21 [[6](#_ENREF_6)] (primers rec_146F/rec_147R) with the forward primer containing an overhanging *Hin*dIII site. Both fragments were cut with *Hin*dIII, ligated and used in a PCR amplifying the complete fragment applying primers rec_148F/rec_149R with overhangs homologous to pSP5. The resulting cassette was recombineered into pSP5 and the selection cassettes subsequently removed. For primer sequences see S1 Table.

***In vitro* functional assays**

COS-7 cells were grown in Dulbecco's Modified Eagle Medium (DMEM) supplemented with 10% fetal bovine serum, 100 U/ml penicillin and 100 μg/ml streptomycin at 37°C and 5% CO_2_ in a humidified atmosphere. Cells were split into 48-well plates (3.8 × 10^4^ cells/well) for enzyme-linked immunosorbent assays (ELISA) to determine cell surface expression or cAMP accumulation assays, into 6-well plates (3 × 10^5^ cells/well) for total ELISA or into 12-well plates (1.2 × 10^5^ cells/well) for IP assays and transfected with Lipofectamine 2000 (Invitrogen) according to manufacturer's protocol. 0.5 µg/well receptor-encoding plasmid DNA/well were transfected for detecting cell surface expression, 1 µg/well for detecting total expression, 0.2-0.5 µg/well for measuring basal activities in cAMP accumulation assays, 1.5 µg/well for measuring basal activities in IP accumulation assays and 0.2 µg/well for analyzing response to peptides. For estimation of cell surface expression, receptors carrying N-terminal HA and C-terminal FLAG tags were analyzed with anti-HA-peroxidase (Roche) in indirect cellular ELISA as described previously [[7](#_ENREF_7)]. For determination of total expression, cells were lysed and receptors were detected using mouse anti-FLAG-M2 (Sigma) and rat anti-HA-peroxidase (Roche) in an indirect cellular ELISA as described previously [[7](#_ENREF_7)]. For measuring cAMP accumulation 48 h post transfection COS-7 cells were washed with serum- and phenol red-free DMEM containing 1 mM 3-isobutyl-methyl-xanthine (IBMX) for 5 minutes. For analysis of agonistic peptides transfected cells were treated with 1 mM peptide. Incubation was stopped by aspirating medium and lysing cells in LI buffer (PerkinElmer Life Sciences). Samples were frozen at −20 °C. To measure cAMP concentration, samples were thawed and the AlphaScreen cAMP assay kit (PerkinElmer Life Sciences) was utilized according to manufacturer's protocol in 384-well white OptiPlate microplates (PerkinElmer Life Sciences) using the Fusion AlphaScreen multilabel reader (PerkinElmer Life Sciences). For IP accumulation assays, transfected COS-7 cells were incubated 24 hours post transfection with 2 μCi/ml *myo*-[^3^H]inositol (18.6 Ci/mmol, PerkinElmer Life Sciences) for 16 hours. Cells were washed once with serum-free DMEM containing 10 mM LiCl and incubated 1 hour either with serum- and phenol red-free DMEM containing 10 mM LiCl. For analysis of agonistic peptides transfected cells were treated with 1 mM peptide. Intracellular IP levels were measured by anion-exchange chromatography as previously described [[8](#_ENREF_8)]. Data obtained from all assays were examined using GraphPad Prism version 5.0 for windows (GraphPad Software).

***In vivo* cAMP measurement**

For determination of cAMP levels in *lat-1* mutant embryos, eggs were collected from a synchronized population of young adult hermaphrodites shortly after fertilization commenced. For measuring cAMP levels upon forskolin stimulation, synchronous cultures of 500 µl pelleted eggs were grown in liquid culture using an overnight culture of OP50 at 3x10^11^cells/ml in S medium. For measuring cAMP levels in adult hermaphrodites upon forskolin stimulation, S medium was supplemented with 80 µM forskolin or 0.8% DMSO, respectively. Cultures were incubated for 48 hours at 22 °C, L4 larvae were purified by flotation on 25 % Ficoll in M9 and collected on 20 µm pore size Nitex nylon filters (Merck Millipore). For measuring cAMP levels in embryos, eggs were collected from adult hermaphrodites either untreated or incubated with 80 µM forskolin or 0.8% DMSO, respectively. Specimen were lysed in a modified LI buffer (5 mM HEPES, 0.1% Tween-20, 1 mM IBMX; pH 7.6) by sonication with 15 30 s pulses in a Bioruptor Standard (Diagenode). Samples were subsequently supplemented with Tween-20 and BSA to a final concentration of 0.2% Tween-20 and 0.1% BSA. 15 µg protein was used for cAMP accumulation measurements with an ALPHAScreen™ (PerkinElmer Life Sciences).

**Fertility Rescue Assay**

Ten L4 hermaphrodites were allowed to lay eggs on separate NGM plates containing 80 µM forskolin or respective solvent and seeded with OP50. Plates were incubated at 22 °C. Every 24 hours hermaphrodites were transferred onto fresh plates until egg-laying ceased and embryos were scored. Experiments were conducted at least in triplicate. Data were examined with an unpaired two-tailed t test for each genotype and condition, means are presented with SD.

**Peptide synthesis**

Solid phase peptide synthesis was performed by standard Fmoc-chemistry on an automated peptide synthesizer MultiPep (Intavis AG). Final side chain deprotection and cleavage from the solid support was achieved using TFA, water and thioanisole (95:2.5:2.5 vol%). Peptides were subsequently purified to > 95% purity by preparative RP-HPLC (Shimadzu LC-8) equipped with a 300x25 mm PLRP-S column (Agilent). For both analytical and preparative use, the mobile phases were water or acetonitrile, respectively, each containing 0.1% TFA. Samples were eluted with a linear gradient of 5% -90% acetonitrile in water: 30 minutes for analytical runs and 90 minutes for preparative runs. Peptide characterization by analytical HPLC (Agilent 1100) and MALDI-MS (Bruker Microflex LT) yielded the expected [M+H]+ mass peaks.

**Generation of transgenic lines**

All transgenic strains with stably transmitting extrachromosomal arrays were generated by DNA microinjection as described [[9](#_ENREF_9),[10](#_ENREF_10)]. Cosmids were injected at a concentration of 1 ng/μl together with the coinjection marker pRF4*[rol-6(su1006)+]* (100 ng/μl) and pBluescript II SK+ vector DNA (Stratagene) as stuffer DNA to achieve a final concentration of 120 ng/μl. DNA was injected into the syncytical gonad of *lat-1(ok1465)/mIn1[mIs14 dpy-10(e128)]* hermaphrodites. Transgenic progeny were isolated and stable lines selected. Multiple independent transgenic lines were established for each transgene tested.

**Supporting References**

1. Dolphin CT, Hope IA (2006) Caenorhabditis elegans reporter fusion genes generated by seamless modification of large genomic DNA clones. Nucleic Acids Res 34: e72.

2. Tursun B, Cochella L, Carrera I, Hobert O (2009) A toolkit and robust pipeline for the generation of fosmid-based reporter genes in C. elegans. PLoS One 4: e4625.

3. Langenhan T, Prömel S, Mestek L, Esmaeili B, Waller-Evans H, et al. (2009) Latrophilin signaling links anterior-posterior tissue polarity and oriented cell divisions in the C. elegans embryo. Dev Cell 17: 494-504.

4. Prömel S, Frickenhaus M, Hughes S, Mestek L, Staunton D, et al. (2012) The GPS motif is a molecular switch for bimodal activities of adhesion class G protein-coupled receptors. Cell Rep 2: 321-331.

5. Petersen TN, Brunak S, von Heijne G, Nielsen H (2011) SignalP 4.0: discriminating signal peptides from transmembrane regions. Nat Methods 8: 785-786.

6. Lee EC, Yu D, Martinez de Velasco J, Tessarollo L, Swing DA, et al. (2001) A highly efficient Escherichia coli-based chromosome engineering system adapted for recombinogenic targeting and subcloning of BAC DNA. Genomics 73: 56-65.

7. Schöneberg T, Schulz A, Biebermann H, Gruters A, Grimm T, et al. (1998) V2 vasopressin receptor dysfunction in nephrogenic diabetes insipidus caused by different molecular mechanisms. Hum Mutat 12: 196-205.

8. Berridge MJ (1983) Rapid accumulation of inositol trisphosphate reveals that agonists hydrolyse polyphosphoinositides instead of phosphatidylinositol. Biochem J 212: 849-858.

9. Mello C, Fire A (1995) DNA transformation. Methods Cell Biol 48: 451-482.

10. Mello CC, Kramer JM, Stinchcomb D, Ambros V (1991) Efficient gene transfer in C.elegans: extrachromosomal maintenance and integration of transforming sequences. Embo J 10: 3959-3970.
