# Supplementary figures and images for: Oriented Cell Division in the C. elegans Embryo Is Coordinated by G-Protein Signaling Dependent on the Adhesion GPCR LAT-1
Source: PLoS Genet. 2015 Oct 27;11(10):e1005624. doi: 10.1371/journal.pgen.1005624 (PMC4624771; doi:10.1371/journal.pgen.1005624)

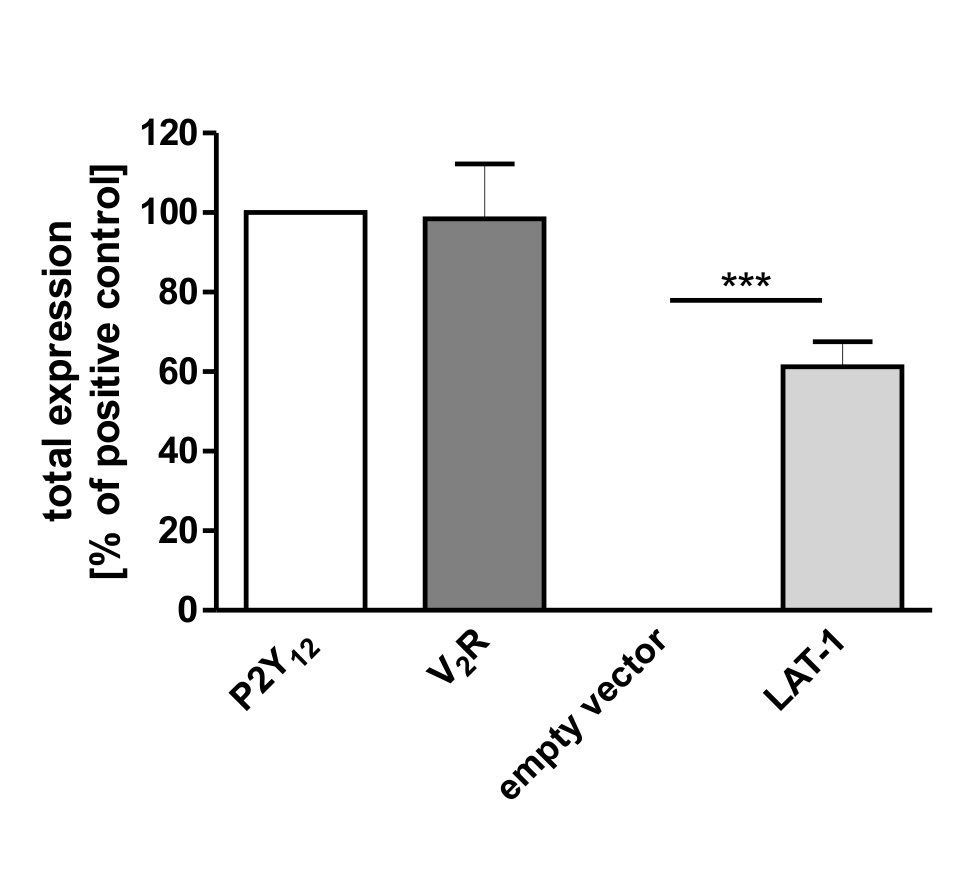

Supplement: S1 Fig — COS-7 cells were transfected with 1 μg of empty vector (pcDps) or plasmid encoding either human ADP receptor P2Y12, the human vasopressin type 2 receptor V2R or LAT-1. Total expression levels were measured 48 hours post transfection using ELISA. Data are displayed as percentage of P2Y12 (positive control) and given as means ± SD of five independent experiments, each performed in triplicate. The non-specific OD value (empty vector) is 0.06 ± 0.01 (set 0%) and the OD value of P2Y12 is 0.92 ± 0.02 (set 100%). *** p < 0.001. (TIF) [file pgen.1005624.s002.tif]

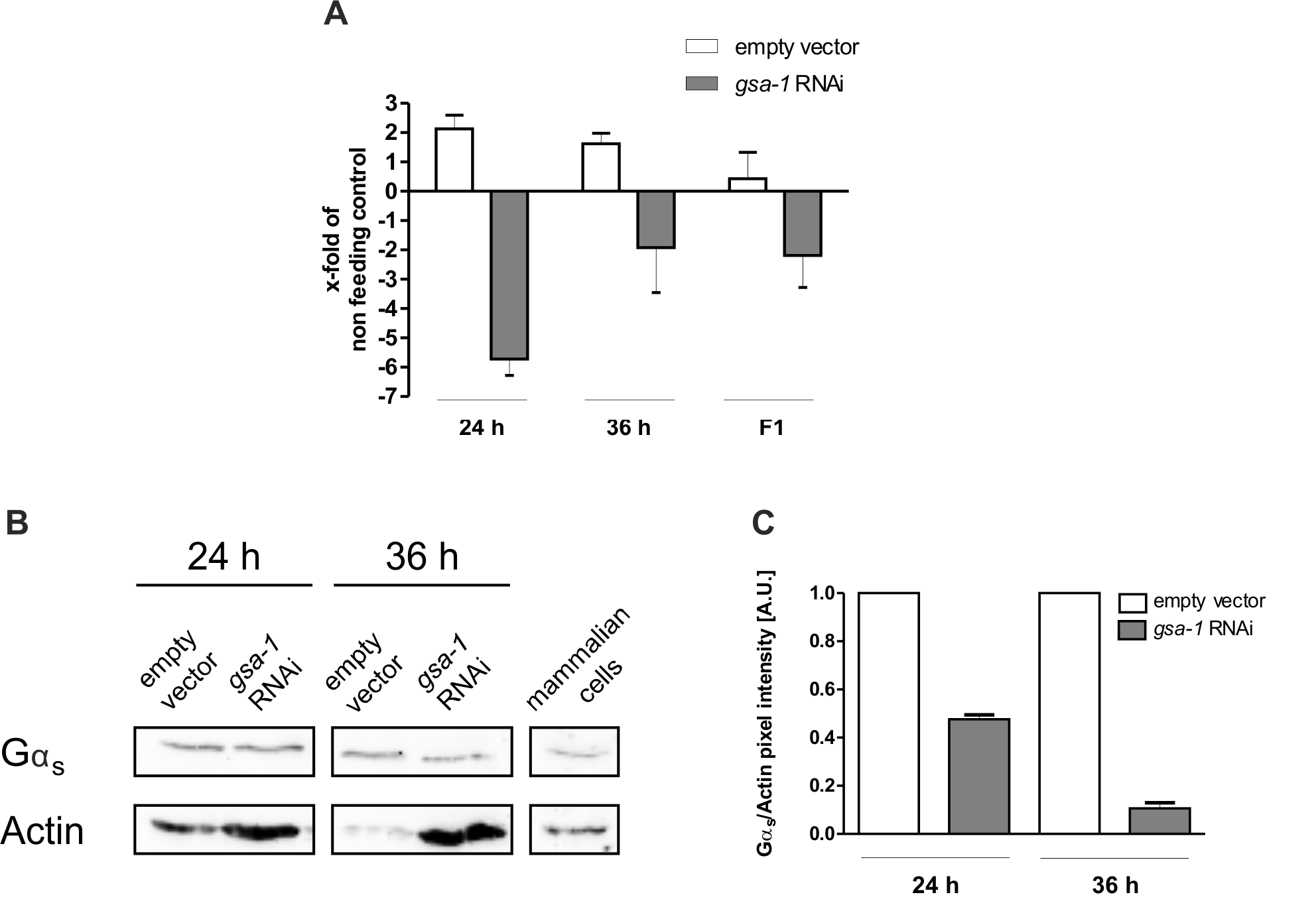

Supplement: S2 Fig — (A) qPCR analysis of gsa-1 in adult wild-type hermaphrodites after 24 hours and 36 hours gsa-1 RNAi and in adult F1 hermaphrodites. Already after 24 hours a knockdown of gsa-1 transcript levels are detected compared to nematodes fed with empty vector (L4440). A complete knockdown is not achieved. Data are normalized using the geometric mean of the reference genes act-1, cdc-42, eif-3 and tba-1 and shown as mean ± SD of three independent experiments each performed in triplicate. (B, C) GSA-1 levels in wild-type hermaphrodites after 24 hours and 36 hours gsa-1 RNAi. Western blot analyses using an anti-Gαs antibody show an increasing reduction of protein with time. The antibody recognizes an epitope sequence which is conserved between mammals and C. elegans and thus, probes mammalian Gαs as well as nematode GSA-1 (B), which are both 46 kDa. HEK293 cells served as mammalian cell control. Actin was used as a loading control (42 kDa). To examine the level of GSA-1 reduction, densitometric analyses of the Western blots were performed (C). Pixels of the different bands and respective backgrounds were quantified and data normalized to the pixel intensity of the respective actin band. Data are given as mean ± SD of three independent experimental analyses. Note that protein of F1 lat-1; gsa-1(RNAi) nematodes was not analyzed due to too little quantities of respective nematodes. (TIF) [file pgen.1005624.s003.tif]

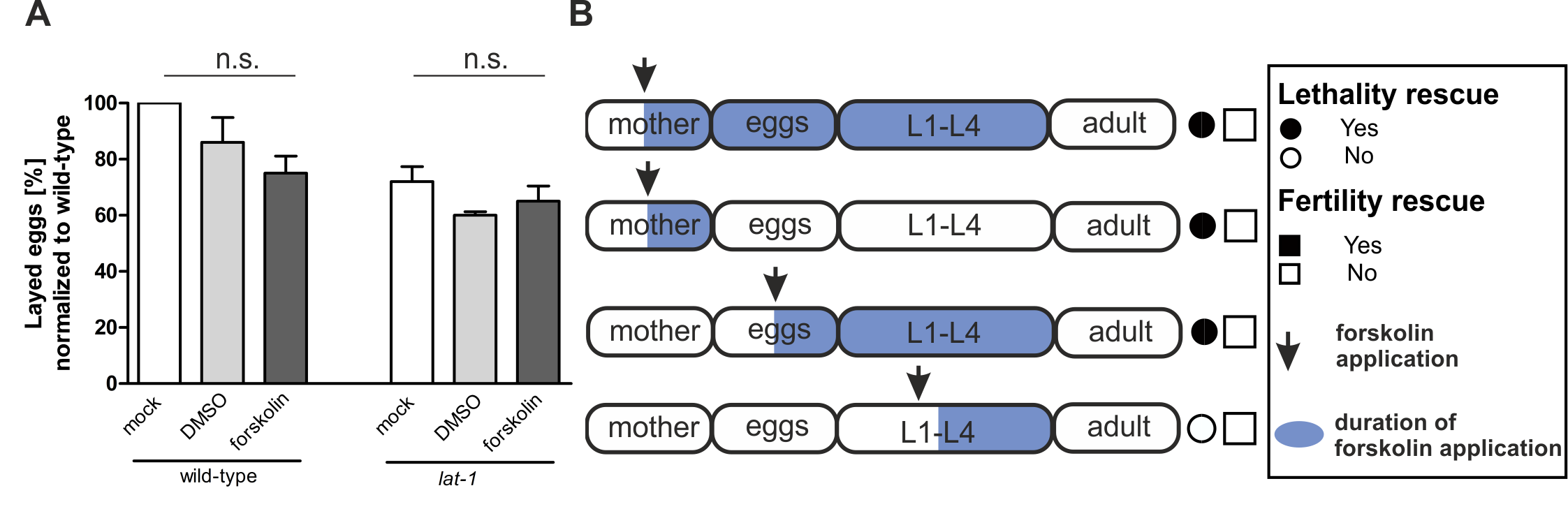

Supplement: S3 Fig — (A) Fertility defects of lat-1 mutants are not rescued by treatment of hermaphrodites from embryonic stages to adulthood with 80 μM forskolin. Brood size was scored compared to untreated (mock), DMSO-treated (0.8%) and wild-type control. Data are given as means ± SD, n.s. not significant. (B) Forskolin treatment (80 μM) does not rescue fertility defects in lat-1 mutants independently of the time point of drug application. Rescue of developmental lethality in lat-1 mutants occurs only when adding forskolin to the mother or the very early embryonic stages. (TIF) [file pgen.1005624.s004.tif]

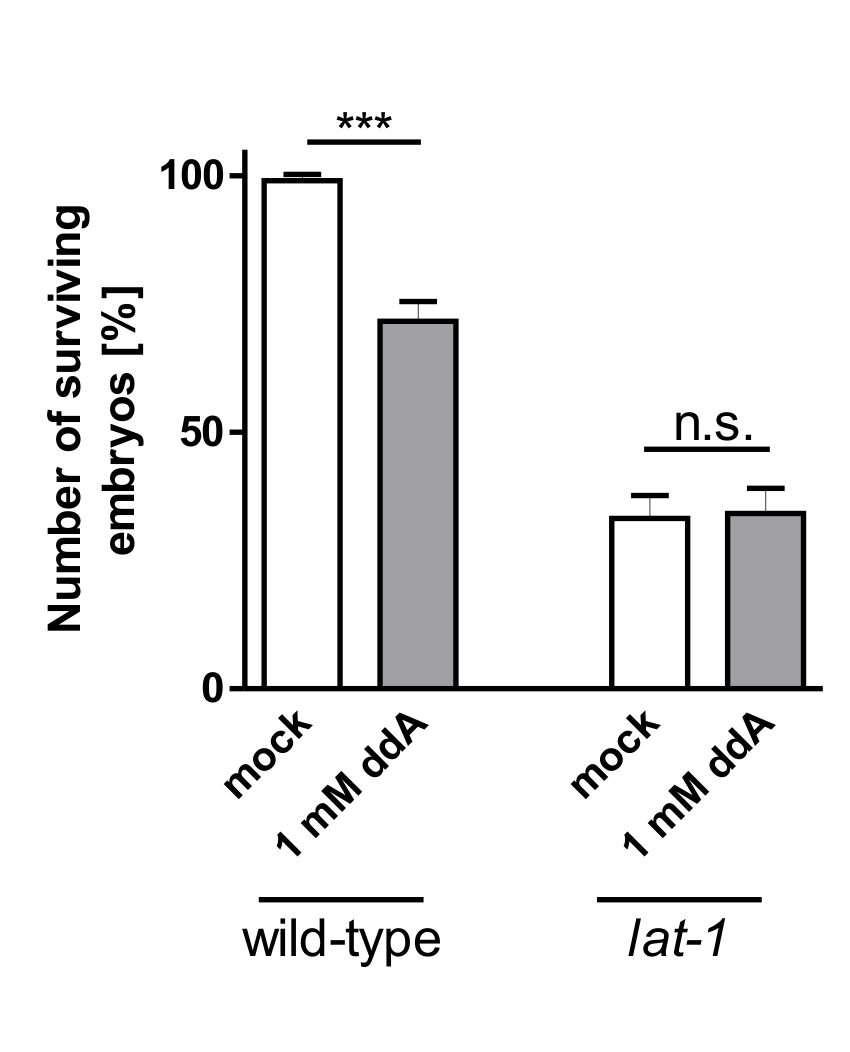

Supplement: S4 Fig — Treatment of adult hermaphrodites and subsequently very early wild-type embryos with the adenylyl cyclase inhibitor 2',5'-dideoxyadenosine (ddA) leads to specific embryonic lethality only observed in wild-type individuals but not in lat-1 mutant embryos. Note that this effect might be not fully penetrant due potential difficulties in drug accessibility. As controls, nematodes were treated with 1% DMSO lacking ddA (mock). Individuals surviving embryogenesis were scored (n > 520). Data are given as means ± SD. n.s. not significant; *** p < 0.001. (TIF) [file pgen.1005624.s005.tif]

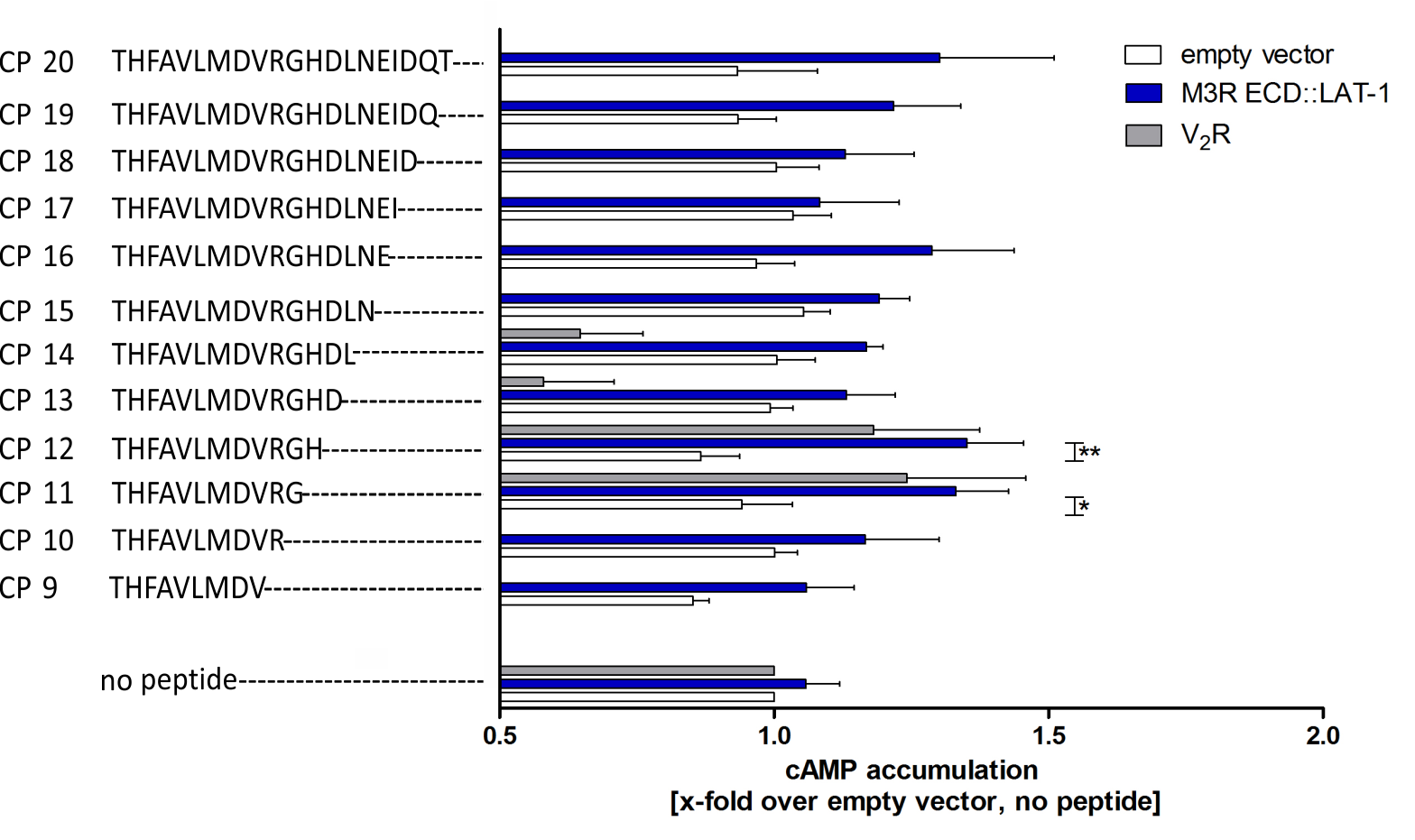

Supplement: S5 Fig — Peptide-stimulated cAMP response of M3R ECD::LAT-1. COS-7 cells transfected with 200 ng empty control vector (pcDps) or plasmid encoding M3R ECD::LAT-1 were stimulated with 1 mM peptide. Subsequently, cAMP levels were measured by cAMP accumulation assay. The human vasopressin type 2 receptor (V2R) served as control for peptide specificity, which does not respond to any of the peptides tested. Basal cAMP levels (empty vector, no peptide) are 5.3 ± 1.3 nM. Data are given as means ± SD of five independent experiments, each performed in triplicate. * p < 0.05; ** p < 0.01. (TIF) [file pgen.1005624.s006.tif]

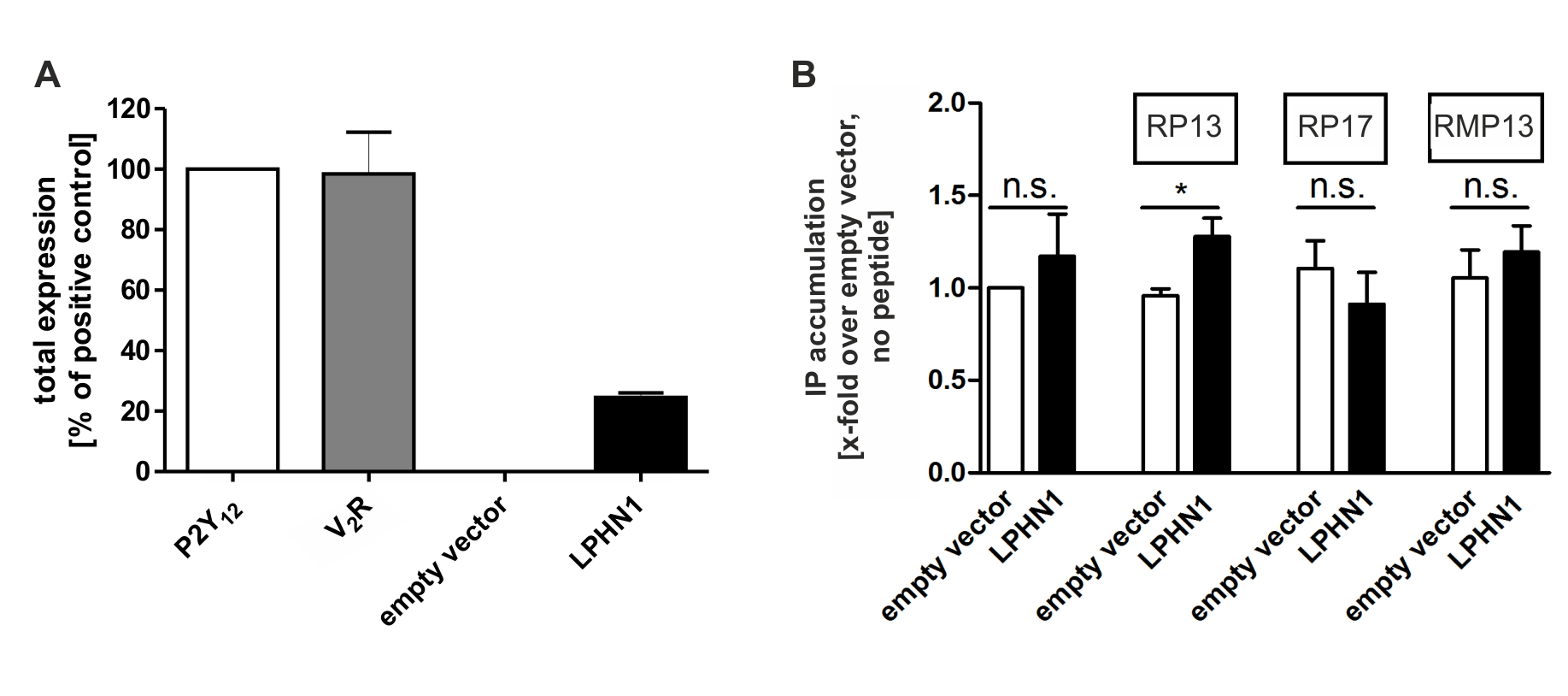

Supplement: S6 Fig — (A) Total levels of LPHN1 in COS-7 cells determined by ELISA. Expression levels were measured 48 hours after transfection with 1 μg of empty vector (pcDps) or plasmid encoding either human ADP receptor P2Y12, the human vasopressin type 2 receptor V2R or rat LPHN1. Data are displayed as percentage of P2Y12 (positive control) and given as means ± SD of five independent experiments, each performed in triplicate. The non-specific OD value (empty vector) is 0.06 ± 0.01 (set 0%) and the OD value of P2Y12 is 0.92 ± 0.02 (set 100%). (B) COS-7 cells transfected with 1.5 μg empty control vector (pcDps) or plasmid encoding LAT-1 were stimulated with 1 mM peptide and IP accumulation assays were performed. An increase in IP levels upon stimulation with peptide RP12 indicates coupling of LPHN1 to a Gq protein. RMP12 served as negative control peptide. Basal IP levels (empty vector, no peptide) are 452 ± 101 CPM/well. Data are normalized to respective non-stimulated controls and are given as means ± SD of two independent experiments, each performed in triplicate. n.s. not significant; * p < 0.05. (TIF) [file pgen.1005624.s007.tif]
